# Resolving cell lineages and gene functions in the developing mouse gastrointestinal tract using in utero transduction

**DOI:** 10.1073/pnas.2614077123

**Published:** 2026-09-09

**Authors:** Ziwei Liu, Krishnanand Padmanabhan, Jingyan He, Katrin Hector, Bettina Semsch, Jia Sun, Viktoria Knoflach, Sarantis Giatrellis, Johan Lorentz, Khachatur Dallakyan, Christian Göritz, Emma Rachel Andersson, Ulrika Marklund

**Affiliations:** ^a^ Department of Medical Biochemistry and Biophysics, Unit of Molecular Neurobiology, Karolinska Institutet SE-171 77, Stockholm, Sweden; ^b^ Department of Cell and Molecular Biology, Karolinska Institutet SE-171 77, Stockholm, Sweden; ^c^ Comparative Medicine, Karolinska Institutet SE-171 77, Stockholm, Sweden

**Keywords:** lineage tracing, in utero transduction, enteric nervous system, gut patterning, neural crest development

## Abstract

How diverse cell lineages emerge and are genetically regulated during organogenesis are central questions in understanding the developmental origins of disease. However, the mouse gut, including its intrinsic enteric nervous system (ENS) derived from migratory neural crest, has remained difficult to experimentally target. Here, we introduce an in utero lentiviral nano-injection strategy that enables early and efficient access to progenitor cells of all major cell types within the developing gut as well as gut-innervating ganglia. Leveraging this approach in combination with DNA barcoding and single-cell transcriptomics, we resolve clonal relationships in all gut lineages, including epithelial, neural, immune, and mesenchymal cell types. Clonal coupling between distinct subsets of fibroblasts and either pericytes, mesothelial cells, or interstitial cells of Cajal, suggested a developmental logic whereby the mesenchymal compartment arises from a set of fate-biased progenitors. Yet, mesenchymal regionalization along the anterior–posterior axis establishes early, whereas the ENS displays broad clonal dispersion across gut regions and acquires subsequent regional identities. We further adapted the platform for temporally controlled cell-type specific gene manipulation and, as a proof-of-principle, show that induced expression of the proneural factor Ascl1 biases ENS progenitor cells toward neuronal differentiation. Together, this work provides insights into refined spatiotemporal lineage relationships within a multigerm-layer organ and establishes a broadly applicable in vivo framework for probing gene function during gastrointestinal and neural crest development.

## Significance

The gastrointestinal tract comprises diverse cell types originating from all three germ layers and includes the neural crest-derived enteric nervous system (ENS). Progress in defining these lineages and their gene regulation is challenged by the limited experimental access to the developing gut. Here, we establish in utero lentiviral transduction as an efficient approach to resolve clonal relationships and address gene functions in defined gut cell types. We show that mesenchyme assumes positional allocation early and differentiates through fate-restricted progenitors, linking specialized mesenchymal cell types to different fibroblasts. In contrast, ENS retains broad competence and acquires regional identities late. Our study provides insights into principles of multi-lineage organogenesis and a framework to dissect the contribution of developmental programs to visceral dysfunction.

The gastrointestinal (GI) tract is essential for life. Congenital gut malformations including Hirschsprung disease and intestinal atresias result in diverse and life-long complications. In the general population, 30% suffer from chronic gut dysfunctions, including disorders of gut–brain interaction (DGBIs) and inflammatory bowel disease (1). Evidence suggests that some of these conditions may originate during fetal development, as premature birth increases the risk of later gut dysfunction (2, 3). Furthermore, congenital chronic intestinal pseudo-obstruction (CIPO) can be myopathic, mesenchymopathic, or neuropathic (4), underscoring the critical role of coordinated interactions among multiple gut cell types in maintaining GI motility. Thus, there is a need to resolve how different cell lineages in the GI-tract develop and integrate during early life.

Experimental gene manipulation can help resolve how gene regulatory networks control cellular differentiation and how developmental deviations can lead to pathology. Owing to the relative ease with which the central nervous system (CNS) can be genetically manipulated using in vivo embryonic transduction or electroporation (5), precise gene regulatory networks controlling central neuronal subtype generation have been revealed (6, 7). These manipulations are possible due to the ventricular structure of the brain and spinal cord, making them accessible to plasmids or viral vectors. In contrast, the internal location of the GI-tract makes it inaccessible to these common approaches.

Normal gut physiology is critically dependent on neuronal control provided by its intrinsic enteric nervous system (ENS), essential for peristalsis, secretion, and blood flow regulation. These functions are modulated by sympathetic innervation, primarily of celiac ganglion neurons, while sensory information is captured from the gut via dorsal root ganglia (DRG) at the trunk regions, and nodose ganglia in cranial regions. The ENS, sympathetic ganglia, and DRG are formed from neural crest cells (NCC), a transient embryonic population originating from the dorsal neural tube (8). In the mouse, delamination of NCC occurs around embryonic day (E) 8 (Theiler stages TS12-13) and thereafter, the migrating cells and the ganglia that form are positioned unfavorably for traditional in utero manipulations that are typically performed from E11.5 (5). Thus, experimental systems to rapidly investigate the development of the murine gut, the ENS, and other gut-innervating ganglia have been lacking.

Recently, we developed neural plate targeting with in utero nano-injection (NEPTUNE) to target the developing CNS at earlier stages (9, 10). With NEPTUNE, ultrasound is used to guide high-titer lentiviral delivery into the mouse amniotic cavity as early as E7.5, allowing access to >80% of CNS cells. The technique can also introduce high-diversity lentiviral barcode libraries to resolve refined lineages using next generation single-cell lineage tracing (11, 12). As distinct barcodes integrate into the nucleus of each transduced cell, and are expressed as RNAs, clonal relationships can be reconstructed after successive cell divisions by single-cell RNA sequencing (scRNA-seq), revealing how individual progenitors contribute to diverse cell types. Here, we asked whether the method can target the GI-tract and neural crest/placodes, whose derivatives innervate the gut, in order to genetically manipulate the developing ENS and reveal clonally linked gut cell types.

We show that ultrasound-guided in utero lentiviral nano-injection at E7.5 transduces progenitors that contribute to the GI tract, including endodermal (i.e., epithelia), mesodermal (e.g., smooth muscle cells, fibroblasts, and interstitial cells of Cajal; ICCs), and ectodermal progenitors (i.e., ENS). In addition, the method efficiently targets extrinsic gut-innervating ganglia (e.g., DRG). By combining high-throughput clonal labeling and transcriptomic profiling of gut cells, we reconstructed lineage relationships across differentiating gut cell types and regions. Of note, we uncovered molecularly defined fibroblast populations that are clonally coupled with either mesothelial cells, pericytes, or ICCs, revealing early diversification paths within the gut mesenchyme. Beyond lineage tracing, we demonstrate the utility of in utero nano-injections for conditional gene perturbation in the ENS. As proof-of-principle we show that timed overexpression of the proneural transcription factor *Ascl1* promotes neuronal differentiation. Taken together, ultrasound-guided in utero nano-injection is a versatile tool to investigate organogenesis and ENS development. Our work offers insight into how neural crest and mesoderm lineages diversify and regionally pattern within the developing gut.

## Results

### Efficient Targeting of Gut-Innervating Ganglia by In Utero Transduction at E7.5.

**Fig. 1.**
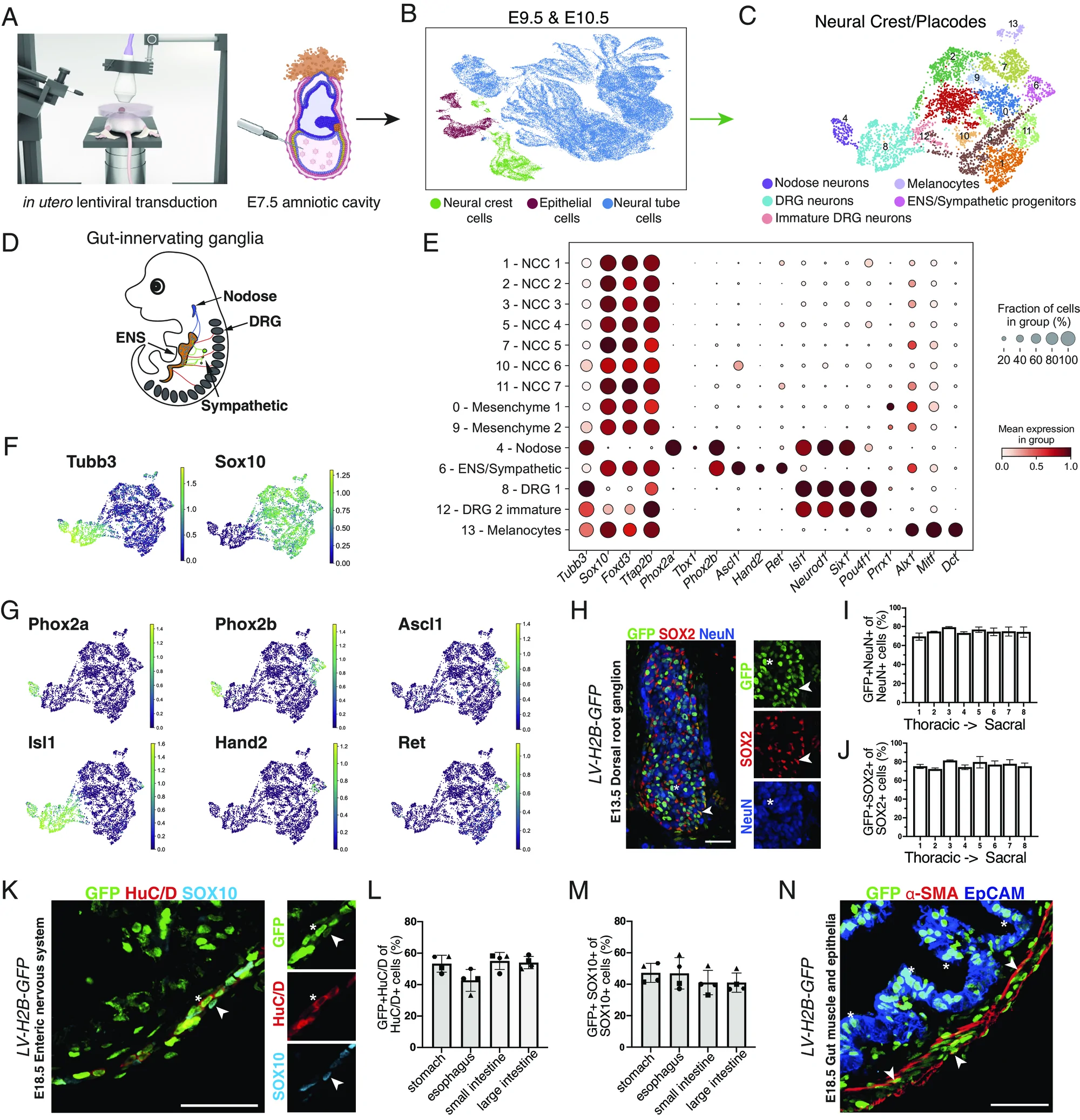
Efficient targeting of neural crest/placode populations destined to innervate the gut by in utero transduction at E7.5. (*A*) Schematic drawing of experimental setup for ultrasound-guided in utero nano-injection. *Left*-most panel reproduced from Mangold et al. (10), with permission (Reproduced from ref. 10, which is licensed under CC BY 4.0.). The *Right* panel was created with BioRender.com. (*B*) Uniform manifold approximation and projection (UMAP) plot of cells recovered from E9.5-10.5 embryos following E7.5 injection (11). (*C*) UMAP plot of reprocessed neural crest/placode cells (green populations in *B*). (*D*) Schematic drawing of neural crest/placode-derived ganglia innervating the gut. (*E*) Dot plot of canonical marker gene expression for neural crest/placode populations across clusters. Dot size represents the fraction of cells expressing the gene and color intensity represents mean expression. (*F*) Feature plots showing expression of neuronal and immature neural crest/placode markers. (*G*) Feature plots showing gene expression associated with nodose ganglia, sympathetic ganglia, DRG and the ENS. (*H*) Representative image of an E13.5 DRG showing GFP expression in SOX2+ neural progenitors (arrowheads) and NeuN+ postmitotic neurons (asterisks). (*I* and *J*) Bar graphs showing proportions of GFP+ NeuN+ neurons and SOX2+ neural progenitors along the thoracic–sacral axis (n = 3 mice). Bars represent mean ± SD. (*K*) Representative image of E18.5 small intestine showing GFP expression in HuC/D+ neurons (asterisks) and SOX10+ ENS progenitors/glia (arrowheads). (*L* and *M*) Bar graphs showing proportion of GFP positive HuC/D+ neurons and SOX10+ progenitors/glia across gut regions (n = 4 mice). Bars represent mean ± SD. (*N*) Representative image of E18.5 gut showing GFP expression in alpha-SMA+ smooth muscle (arrowheads) and EpCAM+ epithelium (asterisks). (Scale bars, 50 µm.)

Lentivirus injection into the amniotic cavity of E7.5 embryos transduces the neural plate and otic placodes (9–11). We therefore hypothesized that gut-innervating ganglia, originating from neural crest or placodes, could also be targeted. We reanalyzed published scRNA-seq datasets from E9.5 and E10.5 embryos, injected at E7.5 with lentiviruses carrying *tdTomato* (11). The cells in this dataset had been broadly categorized as “neural tube cells,” “epithelial cells,” and “neural crest cells” (Fig. 1 *A*–*C* and *SI Appendix*, Fig. S1*A*). To investigate whether developing gut-innervating neurons had been targeted (Fig. 1*D*), we renormalized and subclustered the neural crest cells (4,916 cells), which yielded 14 clusters (Fig. 1 *C* and *E* and *SI Appendix*, Fig. S1*B*). We identified three *Tubb3+* neuronal clusters, while remaining clusters displayed high *Sox10* expression, indicative of neural progenitor or other immature cell states (Fig. 1 *E* and *F* and *SI Appendix*, Fig. S1 *C* and *D*). Among *Sox10*+ clusters, two mesenchymal (*Prrx1+*, *Alx1+*) and one melanocyte (*Mitf*+, *Dct*+) cluster was annotated. Cluster 6 exhibited an autonomic nervous system character (*Phox2b*+, *Hand2+, Ascl1*+) containing presumptive sympathetic (*Isl1+*) and ENS (*Isl1−*) cells (Fig. 1*G*). The remaining *Sox10*+ clusters lacked unique markers and likely represented progenitor or glial cells; these were annotated as NCC1-7 (Fig. 1*E*). Among neuronal clusters, cluster 4 displayed a nodose ganglion identity (*Isl1+*, *Phox2a+, Tbx1+, Tfap2b−*). Cluster 8 and 12 expressed a DRG profile (*Isl1+, Neurod1+, Pou4f1+*), with cluster 12 representing an immature state (higher *Sox10* and *Foxd3* expression) (Fig. 1*E* and *SI Appendix*, Fig. S1 *C* and *D*). We cross-referenced with published spatial transcriptomic data (13) to corroborate marker and cell type localization (*SI Appendix*, Fig. S1*E*).

To further substantiate cluster identities, we addressed Hox expression patterns (*SI Appendix*, Fig. S1 *F* and *G*). Consistent with a cranial origin, the nodose ganglion cluster exhibited anterior Hox expression (e.g., *Hoxa1-4*, *Hoxb1-3*). ENS and parts of the sympathetic ganglia originate from vagal neural crest, and cluster 6 expressed a characteristic suite of *Hoxd3-4* and *Hoxb6* (14). Posterior Hox expression increased at E10.5, particularly within DRG clusters and several NCC clusters, indicative of the anterior-to-posterior emergence of neural crest derivatives. Together, the data demonstrate that E7.5 lentiviral injection targets primordia of gut-innervating ganglia, including ENS, sympathetic ganglia, nodose ganglia, and DRG (Fig. 1*D*).

To confirm that gut-innervating ganglia are persistently labeled beyond E9.5–10.5 (*SI Appendix*, Fig. S2*A*), we performed immunohistochemical analysis of E13.5 DRG and E18.5 gut in embryos injected with lentivirus carrying H2B-GFP at E7.5. Within DRG, both NeuN+ neurons and SOX2+ progenitors/glia were efficiently labeled from thoracic to sacral levels (65 to 83%) (Fig. 1 *H*–*J*). The ENS was uniformly transduced across gut regions, with an average of 51% HuC/D+ neurons and 44% SOX10+ progenitors/glia (Fig. 1 *K*–*M* and *SI Appendix*, Fig. S2 *B*–*E*). In addition, GFP was observed across all gut layers, including in α-SMA+ smooth muscle and EpCAM+ epithelial cells (Fig. 1*N* and *SI Appendix*, Fig. S2*F*). This indicates that viral particles in amniotic fluid not only transduce the neural plate (ectoderm) but are also in contact with endo- and mesodermal cells at E7.5 (*SI Appendix*, Fig. S3 *A* and *B*). Indeed, an early developmental window was crucial for ENS targeting, as injections at E8.0–8.5 failed to label both the ENS and gut epithelium, with only sparse labeling of mesenchymal cells (*SI Appendix*, Fig. S3 *C* and *D*).

Together, these data demonstrate that E7.5 lentiviral injection enables widespread and stable transduction of the GI-tract, encompassing extrinsic gut-innervating neuronal populations, the ENS, and non-ENS gut cell types.

### In Utero Transduction Targets All Major Cell Types in the Developing Gut.

As the injections not only targeted the ENS but the whole gut wall, we reasoned that the approach could be leveraged to determine lineage relationships between gut cell types derived from all three germ layers. Moreover, by capturing different segments of the gut, conclusions could be drawn about the timing of anterior–posterior regionalization. To simultaneously profile the transcriptional identity (cell type) and lineage history (clonality) of single cells, we applied TREX (TRacking and gene EXpression profiling) (11, 12). TREX relies on in utero injection of a high-diversity lentiviral library encoding a 30 N (random nucleotides) barcode and tdTomato resulting in genomic integration into dividing progenitors and stable inheritance of the construct by all descendant cells. The expression of the barcode under a ubiquitous promoter allows its detection by scRNA-seq, enabling reconstruction of clonal relationships among captured cells based on shared barcodes (CloneIDs).

**Fig. 2.**
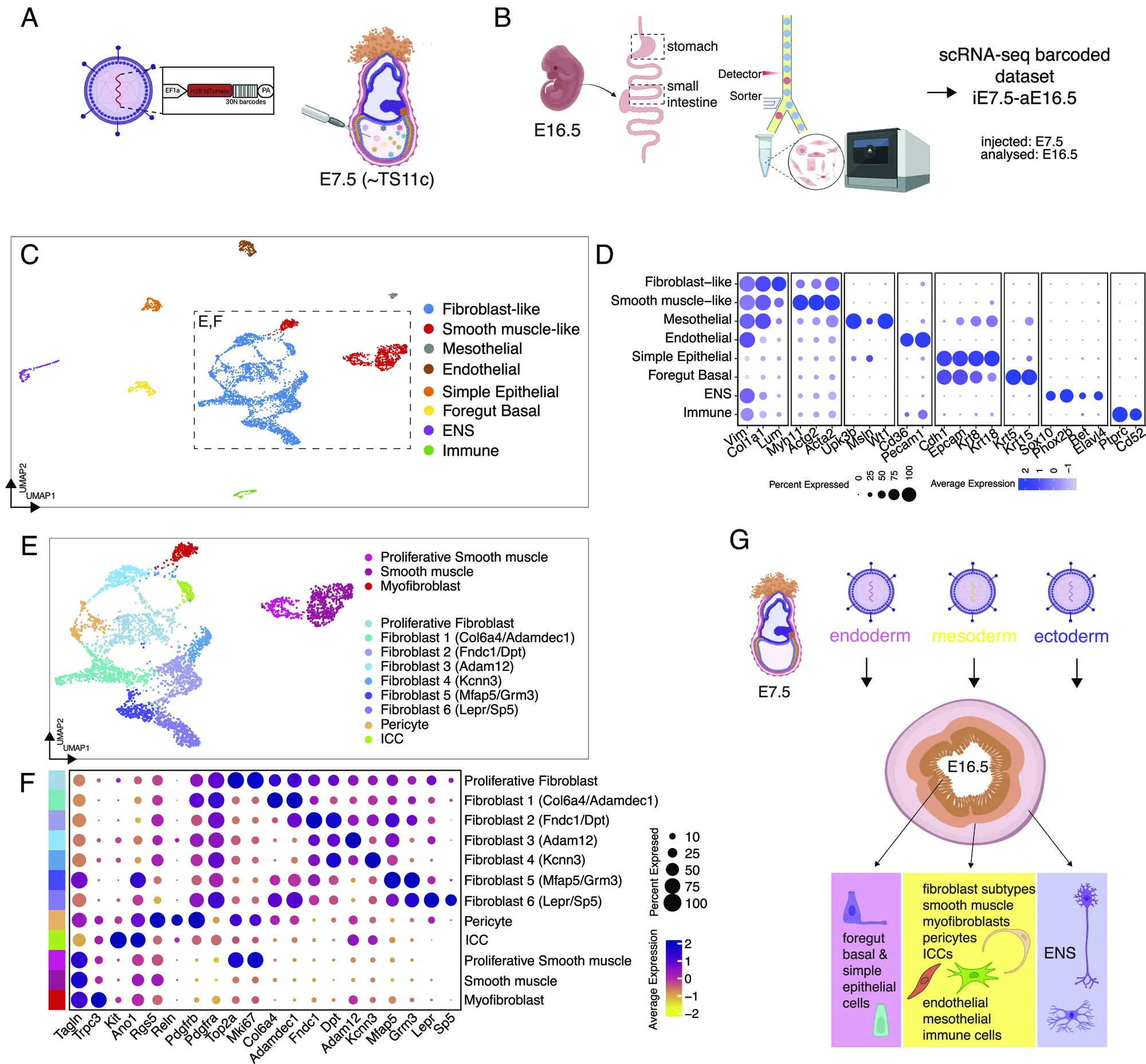
In utero transduction at E7. 5 targets diverse cell types in the developing gut. (*A*) Schematic of injection of lentiviral barcode library into the E7.5 amniotic cavity. (*B*) Experimental workflow to generate the iE7.5–aE16.5 dataset consisting of TOM+ cells, flow sorted from two gut regions. (*C*) UMAP plot showing eight major clusters, annotated based on established marker gene expression. (*D*) Dot plot displaying expression of canonical marker genes used to define major clusters. (*E*) UMAP plot showing subclusters within fibroblast-like and smooth muscle-like populations. (*F*) Dot plot showing gene expression distinguishing fibroblast-like and smooth muscle-like subclusters. (*G*) Schematic drawing of the diverse gut cell types targeted by in utero nano-injection, showing that all three germ layers are efficiently transduced. Part of 2A, B, and K were created with BioRender.com. (*D* and *F*) Dot size represents percentage of cells expressing the gene, and color indicates relative mean expression.

We injected the tdTomato-30 N barcode lentiviral library into E7.5 embryos (Fig. 2*A*) and analyzed at E16.5 (dataset thus called iE7.5–aE16.5). TOM+ cells were isolated from stomach and small intestine and sequenced using the 10× Chromium platform (Fig. 2*B*). After removing low-quality cells and potential doublets, 5,356 cells were retained for analysis (Fig. 2*C* and *SI Appendix*, Fig. S4 *A*–*D*). Cells from both regions were integrated, resulting in eight major clusters defined by canonical lineage markers, corresponding to fibroblast-like cells (*Col1a1+, Lum*+), smooth muscle-like cells (*Myh11+, Acta2+*), endothelial cells (*Pecam1+*), foregut basal cells (*Epcam+, Krt5+*), simple epithelial cells (*Epcam+, Krt5−*), ENS (*Sox10+, Phox2b+, Elavl4+*), immune cells (*Ptprc+*), and mesothelial cells (*Upk3b+, Wt1+*) (Fig. 2*D* and *SI Appendix*, Fig. S4*E*). Further analysis of the simple epithelial cluster revealed enterocytes (*Vil1+*), along with smaller populations of enterochromaffin cells (*Tph1+*), goblet cells (*Clca1+*), and intestinal stem cells (*Lgr5+*) (*SI Appendix*, Fig. S4*F*). The 195 ENS cells formed four clusters representing broad differentiation states. Notably, previously discovered neuronal branches characterized by *Etv1* (Branch A) and *Bnc2* (Branch B) were captured (*SI Appendix*, Fig. S4 *G* and *H*).

Subclustering of the smooth muscle-like cluster revealed a myofibroblast-like state (*Trpc3+, Tagln+*) and a proliferative cluster (Fig. 2 *E* and *F* and *SI Appendix*, Fig. S4 *D* and *E*). Subclustering of the fibroblast-like cluster uncovered additional mesenchymal heterogeneity, including ICC (*Kit+, Ano1+*), pericytes (*Rgs5+, Reln+, Pdgfr*a−) and seven transcriptionally distinct fibroblast subtypes (Fig. 2 *E* and *F* and *SI Appendix*, Fig. S4*E*). These included proliferative fibroblasts (*Top2a+, Mki67+*), Fibroblast 1 (*Col6a4+, Adamdec1+*), Fibroblast 2 (*Fndc1+, Dpt+*), Fibroblast 3 (*Adam12+*), Fibroblast 4 (*Kcnn3+*), Fibroblast 5 (*Mfap5+, Grm3+*), and Fibroblast 6 (*Lepr+, Sp5+*) (*SI Appendix*, Fig. S4 *E* and *I*–*K*). The proliferative fibroblast cluster likely comprised a mixture of all fibroblast-like cells. Collectively, our results demonstrate that in utero lentiviral transduction effectively transduces progenitors of all major gut cell types, encompassing derivatives of the three germ layers (Fig. 2*G*).

### Barcoded In Utero Transduction Reveals Clonal Relationships in the Developing Gut.

**Fig. 3.**
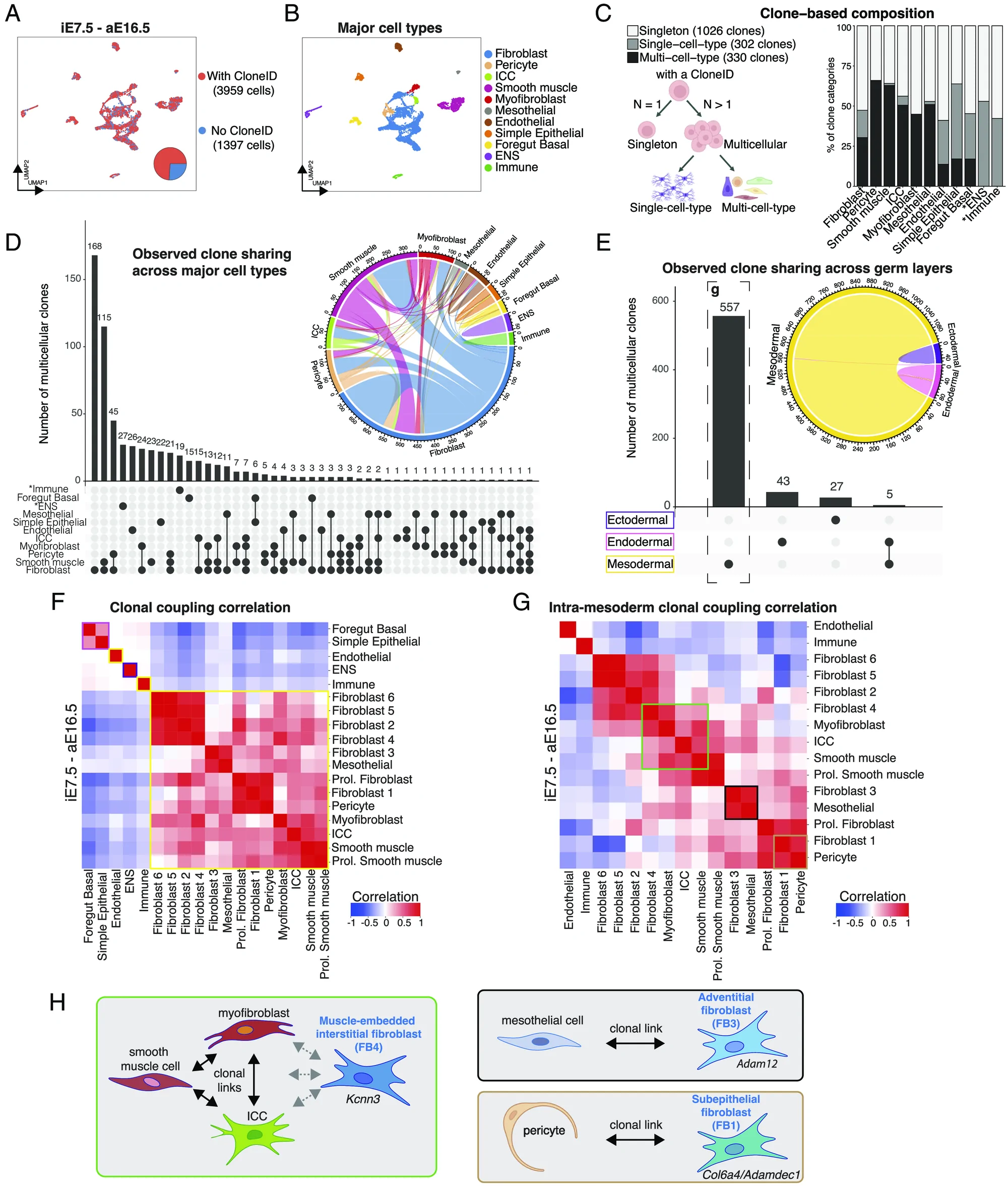
Lentiviral barcoding at E7.5 resolves clonal lineage relations in the developing gut. (*A*) UMAP plot highlighting proportions of cells with CloneIDs in the iE7.5–aE16.5 dataset. (*B*) UMAP plot showing 11 major gut cell types after merging subclusters. (*C*) Stacked bar plot showing distribution of clone categories across major cell types. Schematic drawing describing clonal types (*Left*). (*D*) UpSet plot showing frequencies of clones with different composition of major cell types. (*E*) UpSet plot showing minimal clone sharing across germ layers. *Inset* chord diagrams in (*D* and *E*) illustrate the extent and direction of clonal sharing, with ribbon width proportional to the number of shared clones. (*F* and *G*) Heatmaps showing the correlation matrix of clonal coupling z-scores, computed as deviations of observed clone sharing relative to randomized data, for refined gut cell types (*F*) and restricted to mesoderm-derived cell types (*G*). Colored boxes denote germ-layer origin (pink: endoderm; purple: ectoderm; yellow: mesoderm), or lineage-coupled mesenchymal populations (green: Fibroblast 4 with myofibroblasts, ICC, and smooth muscle; black: Fibroblast 3 with mesothelial cells; brown: Fibroblast 1 with pericytes). (*H*) Schematic summary of lineage relationships among mesenchymal cell types inferred from the clonal coupling analysis. *H* and *Left* panel of *C* were created with Biorender.com.

The comprehensive capture of diverse gut cell types provided excellent conditions to reveal detailed lineage relationships. After barcode extraction, 3,959 cells (73.9%) retained CloneIDs (Fig. 3 *A* and *B* and *SI Appendix*, Fig. S5 *A*–*C*). Cells with or without CloneIDs showed comparable quality metrics and similar CloneID recovery rates were observed across all cell types, indicating unbiased barcode dropout (*SI Appendix*, Fig. S5 *D*–*G*). Most CloneIDs consisted of a single barcode, indicating single lentiviral transductions but up to 4 barcodes were detected in a few cells (0.42% of clones) (*SI Appendix*, Fig. S5*H*).

In total, 1,658 clones were reconstructed and consisted of 1,026 singletons and 632 multicellular clones. The multicellular clones were classified either as single cell–type (302 clones) or multiple cell-types (330 clones), considering major cell type annotations (Fig. 3 *B* and *C*). Multicellular clones contained on average 4.6 cells and maximum 31 cells (*SI Appendix*, Fig. S5*I*). Systematic assessment of multi-cellular clonal compositions per major cell type showed that pericytes, ICCs, smooth muscle, and mesothelial cells almost exclusively consisted of multi-cell-type clones (Fig. 3*C*), suggesting substantial lineage diversification. Instead, ENS and immune cells exhibited only single-cell-type clones, reflecting early lineage restriction of their respective precursors. Clone sharing was predominantly found between mesenchymal cell types in the gut, consistent with their shared mesodermal origin (Fig. 3*D*). However, endothelial and mesothelial cells did not share clones despite sharing clones with other mesenchymal cell types, indicating that their progenitor allocation is distinct at E7.5 (Fig. 3*D*). Cells arising from different germ layers rarely shared clones; only five clones spanned endodermal and mesodermal lineages, likely reflecting residual mesendoderm (15) at the time of injection (Fig. 3*E*).

To systematically resolve lineage relationships among refined cell types, we performed clonal coupling analysis by calculating z-scores for deviations in observed clone sharing relative to randomized data. Hierarchical clustering of Pearson correlations between z-scores segregated cell types into broad groups corresponding to germ-layer origin and revealed intricate clonal coupling relations between mesoderm-derived populations (Fig. 3 *F* and *G* and *SI Appendix*, Fig. S5 *J* and *K*). Notably, ICCs showed strong clonal coupling with smooth muscle cells and myofibroblasts (Fig. 3 *F*–*H*). This is consistent with previous lineage-tracing studies showing that ICCs and myofibroblasts arise from common precursors (16) and that ICCs are not fibroblasts. However, ICCs, myofibroblasts, and smooth muscle cells also showed clonal coupling with Fibroblast 4 (*Kcnn3*+). This fibroblast likely corresponds to *Pdfgra*+ cells of the SIP [smooth muscle cells, ICCs, and PDGFRa (17, 18), recently renamed muscle-embedded interstitial fibroblasts; MIFs (19)] (*SI Appendix*, Fig. S4*I*). Fibroblast 3 (*Adam12+*) showed enriched clonal coupling with mesothelial cells (Fig. 3 *F*–*H*), suggesting an adventitial fibroblast (AF) phenotype, further supported by expression of *Wt1* and *Pi16* (*SI Appendix*, Fig. S4*J*) (20, 21). Fibroblast 1 displayed clonal coupling with pericytes, consistent with the reported localization of *Adamdec1*+ *Col6a4*+ fibroblasts in the subepithelial space (SEFs) (Fig. 3 *F*–*H* and *SI Appendix*, Fig. S4*K*) (22). Clonal coupling analysis thus segregated fibroblast populations into putative contractile, adventitial, and perivascular trajectories which was less apparent from transcriptome-based clustering alone (Fig. 3*H* and *SI Appendix*, Fig. S5*L*).

Clonal coupling patterns among mesoderm-derived populations indicate that mesenchymal lineages diverge early into fate-biased progenitor pools that underpin tissue patterning during gut morphogenesis (Fig. 3*H*). Single-cell lentiviral barcoding at E7.5 thus recapitulates canonical developmental relationships and uncovers early cell fate diversification within the gut, corroborating known lineage relationships while revealing previously unrecognized lineage associations during gut organogenesis.

### Nano-Injection at Early E7.5 Improves ENS Targeting While Revealing Residual Epiblasts.

**Fig. 4.**
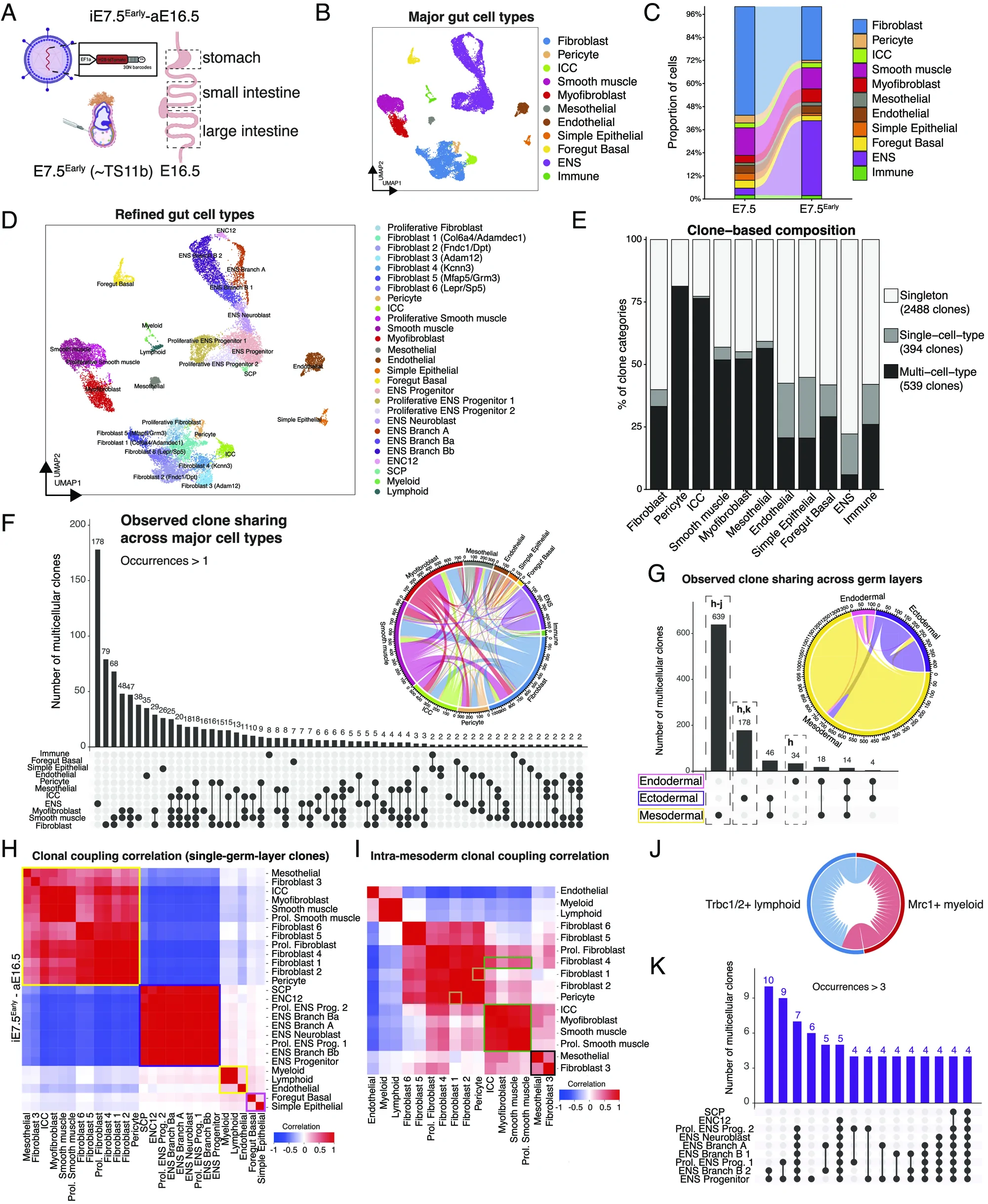
In utero transduction at early E7.5 improves ENS targeting but uncovers presence of epiblast cells. (*A*) Schematic diagram showing the generation of the iE7.5^Early^–aE16.5 dataset consisting of three gut regions. Created with BioRender.com. (*B*) UMAP plot showing major cell types. (*C*) Alluvial plot showing proportion of major cell types in the iE7.5–aE16.5 and iE7.5^Early^–aE16.5 datasets. (*D*) UMAP plot showing refined gut cell types in the iE7.5^Early^–aE16.5 dataset. (*E*) Stacked bar plot showing distribution of clone categories across major cell types. (*F*) UpSet plot showing frequencies of clones with different composition of major cell types. (*G*) UpSet plot showing notable clone sharing across germ layers. *Inset* chord diagrams in (*F* and *G*) illustrate the extent and direction of clonal sharing (*H* and *I*). Heatmaps showing the correlation matrix of clonal coupling z-scores for refined gut cell types (*H*) and restricted to mesoderm-derived cell types (*I*). Colored boxes denote germ-layer origin (pink: endoderm; purple: ectoderm; yellow: mesoderm), or lineage-coupled mesenchymal populations (green: Fibroblast 4 with myofibroblasts, ICC, and smooth muscle; black: Fibroblast 3 with mesothelial cells; brown: Fibroblast 1 with pericytes). (*J*) Chord diagram showing self- and cross-linking between Mrc1+ myeloid and Trbc1/2+ lymphoid cells. (*K*) UpSet plot showing co-occurrence of ENS cell states/types in multicellular clones.

Attempting to enrich for ENS targeting, we performed another nano-injection of the barcode lentiviral library aimed at an earlier developmental stage (E7.5^Early^, ~TS11b; *SI Appendix*, Fig. S3 *A* and *B*), reasoning that primordia of neural crest could be physically more accessible and remain for a longer time before delamination. To gain further information about gut regionalization, we collected stomach, small intestine, and large intestine at E16.5 to generate the iE7.5^Early^–aE16.5 dataset (Fig. 4*A*). Data were processed similarly as in the previous experiment, and the same major cell types were recovered (Fig. 4*B* and *SI Appendix*, Fig. S6 *A*–*E*). Notably, the proportion of ENS cells was increased by 10-fold (Fig. 4*C*), making ENS the most abundant cell population, and corroborating greater lentiviral accessibility to neural crest primordia at this earlier stage of injection. Higher-resolution clustering further resolved subpopulations within immune and ENS lineages, revealing myeloid (*Mrc1*+, *Cd68*+, *C1qc+*) and lymphoid (*Trbc1/2*+) subpopulations, lymphatic signatures (*Lyve1*+, *Pecam1*+), refined ENS neuron divisions, and Schwann cell precursors (SCPs) (Fig. 4*D* and *SI Appendix*, Fig. S6 *F*–*I*).

CloneIDs could be assigned to 77.7% of the 21,749 cells (*SI Appendix*, Fig. S7 *A*–*E*), yielding 3,421 clones comprising 2,488 singletons, and 933 multicellular clones, consisting of 394 single-cell-type clones, and 539 multi-cell-type clones (Fig. 4*E*). The average and maximum size of multicellular clones were larger in the E7.5^Early^–aE16.5 dataset (*SI Appendix*, Fig. S7*F*). Consistent with the first experiment, mesenchymal populations comprised mainly of multi-cell-type clones, whereas ENS, immune and epithelial cell populations were more lineage-restricted (Fig. 4*E*). Although most multicellular clones were composed of a single germ layer, a notable fraction (~9%) of clones spanned more than one germ layer derivative (~1.5% with three germ layers; 14 clones) (Fig. 4 *F* and *G*). These cross-germ-layer clones suggest the presence of pluripotent epiblast cells at E7.5^Early^, in agreement with recent spatial transcriptome analyses of murine gastrula (23). Even after down-sampling this second dataset (iE7.5–aE16.5^Early^) to match the clone and cell number composition of the first dataset (iE7.5–aE16.5), cross-germ-layer clones involving the ENS or immune cells were consistently detected in the vast majority of iterations (999/1,000 and 1,000/1,000, respectively; *SI Appendix*, *Methods*), indicating that earlier viral transduction, rather than dataset size, accounts for their recovery.

The presence of cross-germ-layer clones in the iE7.5^Early^–aE16.5 dataset complicated lineage analysis between refined cell types, but we reasoned that analysis using germ-layer pure clones would still be relevant. Clonal coupling analysis of pure clones (851 clones) recapitulated patterns observed in the iE7.5–aE16.5 dataset (compare Fig. 3*F* with Fig. 4*H*; *SI Appendix*, Fig. S7*G*). We next restricted analysis to pure mesoderm clones (639 clones) (Fig. 4*I* and *SI Appendix*, Fig. S7*H*). As previously observed (Fig. 3 *G* and *H*), ICCs, myofibroblasts, and Fibroblast 4 (putative MIFs) featured stronger clonal coupling, as did mesothelial cells and Fibroblast 3 (putative AFs) (Fig. 4*I*). Although Fibroblast 1 (putative SEFs) was not adjacent to pericytes in the correlation heatmap, it exhibited the strongest clonal coupling to pericytes of all fibroblasts (Fig. 4*I* and *SI Appendix*, Fig. S7*H*). Within the immune population, *Mrc1*+ myeloid and *Trbc1/2*+ lymphoid cells shared few clones, indicating early lineage specification prior to gut colonization (Fig. 4*J*). The large ENS dataset allowed detailed clonal analysis of ENS subclusters, revealing that many clones consisted of multiple ENS states/types (Fig. 4*K*). Notably, SCPs showed clonal coupling with all ENS subtypes, however less so with the more mature neuron clusters ENC12 and Branch Bb which may differentiate before SCP arrival to the gut (*SI Appendix*, Fig. S7*G*, gray box). Coupling was strong between Branch A and B neurons (Fig. 4*K* and *SI Appendix*, Fig. S7*G*), suggesting no predetermination of Branches in agreement with an earlier lineage-tracing study (24).

Together, our results from the E7.5^Early^ injection highlights the importance of precise embryonic staging for enriched targeting of specific lineage precursors. The optimal window for ENS targeting appears to coincide with early E7.5 (~TS11b stage), when some epiblast cells are still present. This stage is therefore advantageous for efficient and comprehensive ENS targeting, while posing challenges for lineage analysis.

### Clonal Sharing between Gut Segments Reveals Lineage-Specific Timing of Regional Identity Acquisition.

We combined our regionally resolved scRNA-seq datasets and analyzed transcriptional similarities and differences across GI regions. While most cell types and gene expression patterns were consistent across all regions, we uncovered some regionally distributed cell types, expression patterns, and fibroblast states that may contribute to establishing functional specialization of GI regions (*SI Appendix*, Fig. S8).

**Fig. 5.**
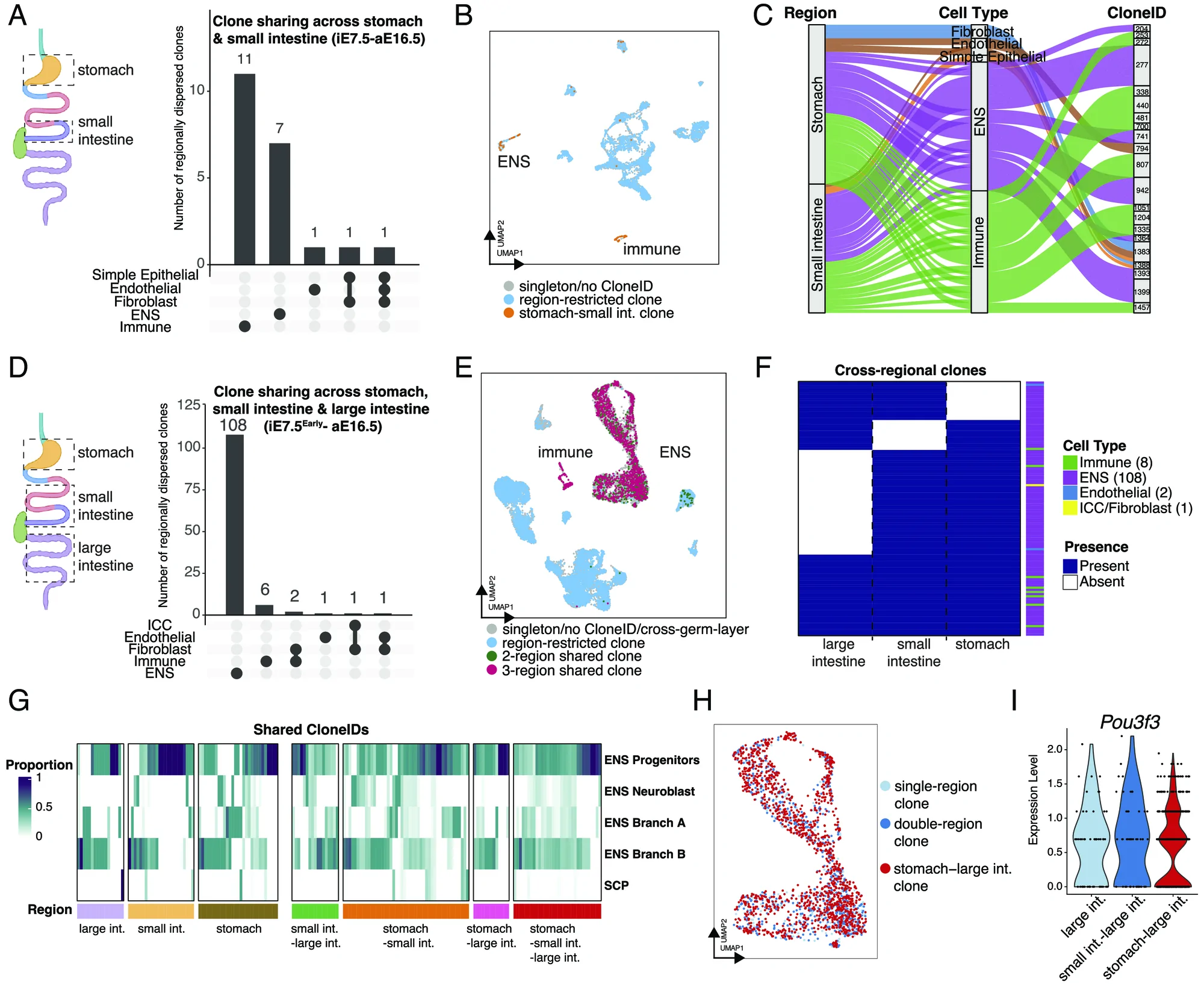
Regional clone sharing reveals long-range ENS migration and early regional patterning of mesenchymal populations along the GI tract. (*A*) UpSet plot showing cross-region clones in the iE7.5–aE16.5 dataset. (*B*) UMAP plot highlighting cells in cross-region and local clones. (*C*) Alluvial plot illustrating relationship between tissue region, cell type, and CloneID of the cross-region clones illustrated in (*A*). (*D*) UpSet plot showing cross-region clones in the iE7.5^Early^–aE16.5 dataset (restricted to single-germ-layer clones). (*E*) UMAP plot highlighting cells in cross-region and local clones. (*F*) Heatmap showing the regional distribution of clones across gut regions in the iE7.5^Early^–aE16.5 dataset, with binary indication of presence or absence. Colored bars denote cell type identities of clones. (*G*) Heatmap showing clonal composition of ENS cell states/types for cross-region or local clones, revealing no apparent bias. (*H*) UMAP plot highlighting the differentiation state of ENS clones with different dispersion. (*I*) Violin plot showing comparable levels of *Pou3f3* expression in colonic ENS cells that are local or shared CloneIDs with cells in the small intestine and/or stomach. Parts of *A* and *D* were created with Biorender.com.

Having resolved lineage relationships between and within gut cell types, we next examined the regional dispersion of clones across the upper and lower GI tract. In the iE7.5–aE16.5 dataset, 21 clones linking the stomach and small intestine were identified. Of these, 18 clones consisted exclusively of ENS or immune cells, accounting for 25.9% of ENS and 57.9% of immune clones (Fig. 5 *A–C*). These regional dispersions are consistent with the extrinsic origins and migratory behavior of these lineages: ENS precursors migrate along the entire gut axis (8), while the first wave of immune cells originates from the yolk sac and subsequently colonizes the gut (25, 26). Clones corresponding to other cell types were predominantly restricted to a single gut segment, suggesting that most endoderm- and mesoderm-derived progenitors had acquired their regional identities by E7.5 (~TS11c).

Considering single-germ-layer clones in the iE7.5^Early^–aE16.5 dataset, we identified 119 region-spanning clones, again mainly from ENS and immune lineages, accounting for 60.7% of ENS and 66.7% of total immune clones (Fig. 5 *D–F*). Epithelial or fibroblast clones did not span regions, indicating that their progenitors are regionally patterned also at slightly earlier gastrula stages. We next examined the ENS subtype composition within cross-regional and local clones and found them to be similar. Notably, both Branch A and B neurons were present in local and cross-regional clones, consistent with a broad developmental competence among ENS progenitors during embryogenesis (Fig. 5*G*). A distinguishing feature of colonic ENS is *Pou3f3* (27), while it has not been shown if expression is induced in vagally derived enteric *Pou3f3*− precursors as they enter the large intestine. Our barcoding experiment allowed us to clonally link stomach-resident ENS (derived exclusively from the vagal neural crest) to *Pou3f3+* cells in the large intestine. We found that stomach/large intestine clones distributed randomly among ENS differentiation stages and neuron identities (Fig. 5*H*). Large intestine cells within large intestine-only clones and stomach/large intestine clones showed similar expression frequency and level of *Pou3f3* (Fig. 5*I*). Together, these findings support that colonic ENS derived from vagal neural crest acquire a colon-specific identity by responding to local cues. To further validate the observed regional patterning of the gut, we generated and analyzed a third dataset representing targeting of an even earlier stage than the second dataset, containing a twofold enrichment of ENS (named iE7.5^Early2^–aE16.5). This analysis confirmed the broad developmental potential of ENS, its cross-regional dispersion and regionally imposed colon-specific expression pattern (*SI Appendix*, Fig. S9).

Finally, to explore lineage relationships among gut-innervating ganglia, we examined clonal barcode sharing within the E9.5–E10.5 dataset (Fig. 1). The nodose cluster showed no barcode sharing with any other cluster, whereas the two DRG clusters exhibited strong coupling to one another but minimal coupling to ENS/sympathetic cells. These findings support an early regional segregation at E7.5 between progenitor populations destined to form distinct gut-innervating ganglia (*SI Appendix*, Fig. S10). Although larger datasets will be required for definitive conclusions, this analysis demonstrates the potential of early embryonic barcoding to resolve clonal relationships among peripheral ganglia.

### Cre-dependent Lentiviral Cargo Enables Cell Type–Specific Expression.

Progenitors of essentially all gut cell types are transduced by in utero lentiviral nano-injection at E7.5. For applications requiring cell type–specific targeting, this approach therefore needs adaptation. We previously showed that Baf53b-Cre specifically labels neurons in the juvenile ENS (28). To investigate its utility during embryonic development, Baf53b-Cre;R26-tdTomato embryos were analyzed at two stages (*SI Appendix*, Fig. S11 *A* and *B*). Reporter expression was restricted to HuC/D+ neurons and absent from SOX10+ progenitor/glial cells. We found that ~50% of HuC/D+ neurons expressed TOM at E14.5, increasing to ~90% at E18.5. Baf53b-Cre activity is thus confined to differentiating neurons, with a slight delay relative to HuC/D expression.

**Fig. 6.**
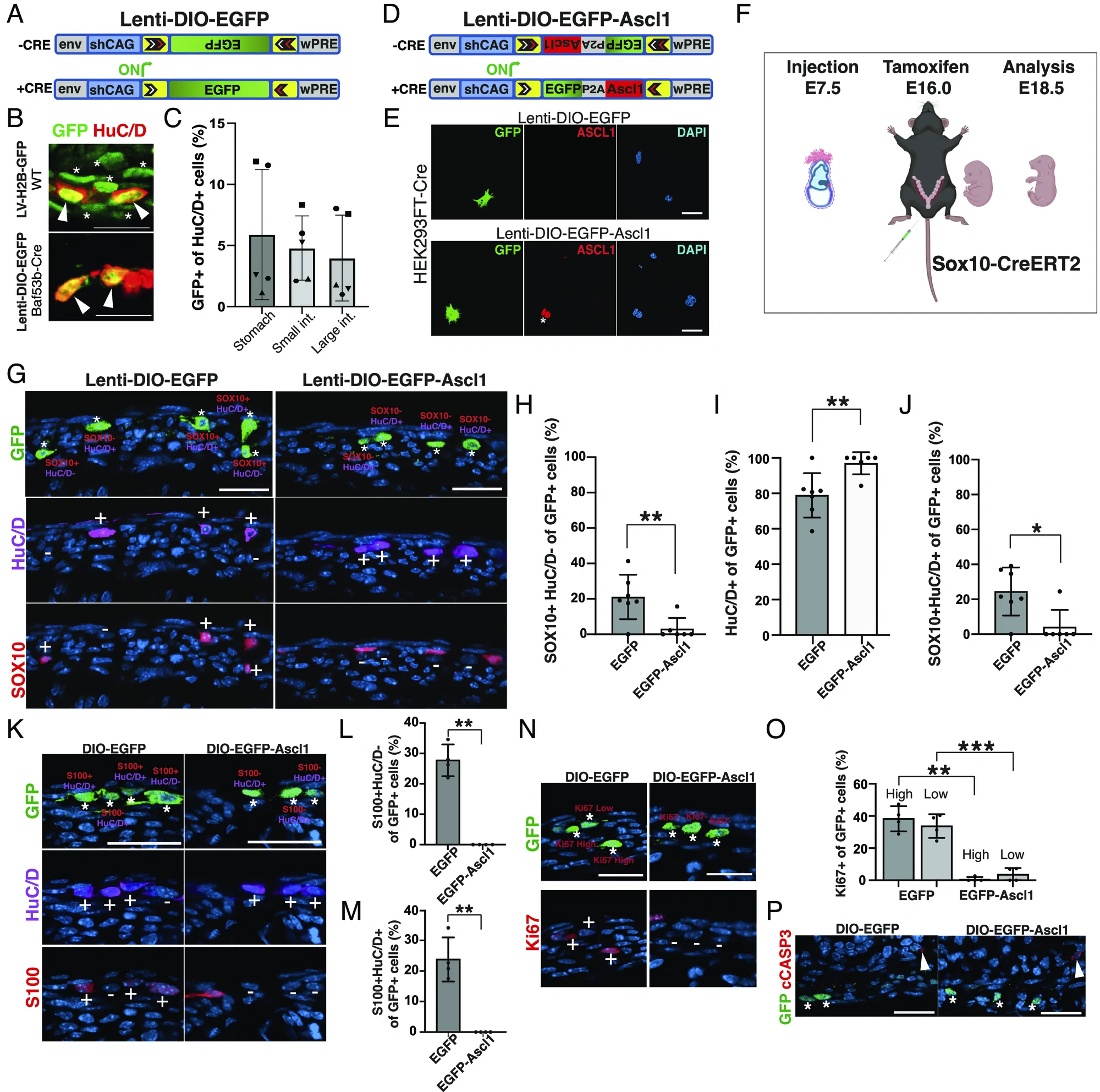
Temporal overexpression of *Ascl1* in Sox10+ cells promotes neuronal differentiation. (*A*) Schematic overview of the Lenti-DIO-EGFP vector, before and after Cre-mediated gene inversion. (*B*) Immunohistochemistry of intestine from wild-type mice injected with LV-H2B-GFP showing GFP expression in both HuC/D− (asterisks) and HuC/D+ (arrowheads) cells, and Baf53b-Cre mice injected with Lenti-DIO-EGFP showing GFP expression restricted to HuC/D+ cells (arrowheads). (*C*) Bar graph showing the percentage of HuC/D+ cells expressing GFP across gut regions in Baf53b-Cre embryos injected with Lenti-DIO-EGFP (n = 5 mice). (*D*) Schematic drawing of the Lenti-DIO-EGFP-Ascl1 construct with and without Cre-mediated gene inversion. (*E*) Representative immunohistochemistry images showing validation of transgene expression in Cre-expressing HEK293FT cells. (*F*) Schematic of experimental procedure for in vivo overexpression of EGFP-Ascl1 or EGFP in ENS progenitors of Sox10-CreERT2 embryos. Created with BioRender.com. (*G*) Representative images of transduced E18.5 small intestine. (*H*−*J*) Bar graphs showing the percentage of GFP+ cells expressing selected markers. (*K*) Representative images of transduced E18.5 small intestine showing glial marker expression and corresponding bar graphs in (*L* and *M*) displaying percentages in GFP+ cells. (*N*) Representative images of E18.5 intestine depicting Ki67 expression with bar graphs in (*O*) showing percentages in GFP+ cells. (*P*) Representative images showing lack of cCASP3 in GFP+ cells (169 GFP+ cells analysed in each condition from three mice). Arrows depict cCASP3 staining in non-GFP cells. Asterisks in (*G*, *K*, *N*, *P*) denote GFP+ cells and text indicate their status of marker expression. All bars represent mean ± SD. (*H*) **P = 0.0086; (*I*) **P = 0.0086; (*J*) *P = 0.011, (*L*) **P = 0.0018; (*M*) **P = 0.0071; (*O*; high) **P = 0.0019, (*O*; low) ***P = 0.0004. n = 4 to 7 mice per condition. WT: wild-type. [Scale bars, 20 µm. Blue in (*G*, *K*, *N*, and *P*) shows DAPI staining.] WT: wild-type. (Scale bars, 20 µm.)

To achieve conditional targeting with in utero nano-injection, we designed a lentiviral vector expressing an inverted double-floxed EGFP reporter, Lenti-DIO-EGFP (Fig. 6*A*). Injection of Lenti*-*DIO-EGFP into Baf53b-Cre mice resulted in restricted GFP labeling of HuC/D+ neurons (~4 to 6%), in contrast to LV-H2B-GFP which additionally labels non-ENS cells (Fig. 6 *B* and *C*). In utero nano-injection of conditional lentiviral vectors thus enables precise targeting of specific gut cell types.

### Timed Overexpression of Ascl1 Promotes Neuronal Differentiation of ENS Progenitors.

Having validated that lentiviral DIO-EGFP expression can be controlled by Cre, we next investigated the possibility to temporally control gene expression in ENS progenitor cells. Sox10-CreERT2 mediates efficient, temporally controlled recombination in ENS progenitors (14, 24, 29). To assess the efficiency and kinetics of Cre-dependent recombination, tamoxifen was administered to Sox10-CreERT2 females mated with R26-EYFP males at E16.5. 26 h later, ~50% of SOX10+ cells expressed EYFP and ~11% of HuC/D+ neurons, indicating efficient and rapid labeling of progenitors/glia and a subset of newly differentiated neurons (*SI Appendix*, Fig. S11 *C*–*E*). As a proof-of-principle, we sought to overexpress *Ascl1*, a proneural transcription factor essential for enteric neurogenesis, as demonstrated by us and others (30, 31). We generated a Cre-inducible *Lenti-DIO-EGFP-Ascl1* construct, in which *Ascl1* was linked to EGFP via a self-cleaving P2A peptide (Fig. 6*D*). Cre-dependent ASCL1 expression was validated using a HEK293FT-Cre cell line (Fig. 6*E*). We overexpressed ASCL1 at late embryonic stages, when both glio- and neurogenesis are ongoing, to assess its ability to promote neuronal differentiation. Sox10-CreERT2 embryos were injected at E7.5 with lentivirus carrying either DIO-EGFP or DIO-EGFP-Ascl1, followed by tamoxifen administration at E16.0 and analysis at E18.5 (Fig. 6*F*). As compared to controls, ASCL1 overexpression reduced the EGFP+ progenitor/glia population (SOX10+ HuC/D−) from ~20 to ~3% (Fig. 6 *G* and *H*) and increased the EGFP+ neuronal population (HuC/D+) from ~80 to ~97% (Fig. 6 *G* and *I*). Notably, ~25% of EGFP+ cells in controls coexpressed SOX10 and HuC/D, indicative of a transitional population, which was reduced to ~4% upon ASCL1 overexpression (Fig. 6 *G* and *J*). The enteric glia/mature progenitor marker S100 was also repressed by ASCL1 overexpression, reducing the proportion from ~25% in control EGFP cells (both HuC/D+ and HuC/D−) to 0% in ASCL1-EGFP cells (Fig. 6 *K*–*M*). Associated with enhanced neurogenesis, ASCL1 overexpression led to a pronounced decrease in proliferation as both Ki67^high^ and Ki67^low^ GFP cells were reduced (each category from >30 to <4%) (Fig. 6 *N* and *O*), while no effect on cell survival was evident (Fig. 6*P*).

Together, these findings demonstrate that ASCL1 overexpression during a gliogenic window is sufficient to bias enteric progenitors toward neuronal differentiation. More broadly, this approach establishes ultrasound-guided in utero delivery of Cre-inducible lentiviral constructs as a powerful method for temporally controlled, cell type–specific gene manipulation in developing gut.

## Discussion

Our understanding of gut development and related diseases has advanced substantially through recent technologies, including scRNA-seq and spatial transcriptomics (27, 28, 32). However, methods to define lineage relationships and interrogate gene function in vivo have lagged behind, partly due to the inaccessibility of embryonic gut using traditional targeting methods. Here, we present ultrasound-guided in utero nano-injection as an efficient methodology to access major gut cell populations in mouse embryos, including epithelial and mesenchymal populations, the ENS, as well as gut-extrinsic ganglia. Combined with high-diversity lentiviral barcoding, this approach establishes a versatile lineage-tracing platform, and coupled with Cre-dependent constructs, the approach supports temporally controlled, cell type–specific gene manipulation in the developing gut.

Barcode-based lineage tracing combined with scRNA-seq overcomes key limitations of traditional Cre-based lineage-tracing approaches as they depend on targeting stem cells defined by single marker genes with often equivocal specificities that risk confounding interpretation. In contrast, the method presented here is marker-independent and enables comprehensive reconstruction of lineage relationships. It allowed us to distinguish transcriptionally related mesenchymal populations and refine their cellular identities, lineage relationships, and early development. First, we identified three fibroblast states with distinct clonal relationships to specialized mesenchymal cell types. *Kcnn3*+ fibroblasts (putative MIFs) were clonally related to ICCs and myoblast/smooth muscle cells, suggesting shared progenitors. *Adamdec1*+/*Col6a4+* fibroblasts (putative SEFs) were linked to pericytes, whereas *Adam12*+/*Wt1+* fibroblasts (putative AFs) were clonally related to mesothelial cells. Notably, no consensus nomenclature currently exists for gut fibroblasts. Clonal analysis therefore provided important orthogonal support for subtype assignments and supported a developmental logic whereby stromal gut compartments are assembled by a set of fate-biased mesenchymal progenitor populations. Second, by analyzing clonal dispersion across gut regions, we demonstrate that gut mesoderm is already regionally specified along the anterior–posterior axis by E7.5. Third, we present regional diversity among mesenchymal cell types, likely reflecting early programs that shape region-specific gut wall composition. Overall, these findings highlight how lineage information complements transcriptomic and anatomical analyses to better define gut stroma development. Increasing evidence shows that different fibroblast subpopulations may have distinct roles in cancer, inflammation, and fibrosis (33), underscoring the importance of better understanding their heterogeneity and development.

By integrating multiple gut regions, we found that region-spanning clones were common in migratory lineages. Within the ENS, clonal sharing across stomach and colon supports a model in which vagal progenitors acquire region-specific features, such as colonic *Pou3f3* expression, in response to local cues. In contrast, the restriction of epithelial and mesenchymal clones to single regions indicates that regional identity of these compartments is established early. Together with the region-specific transcriptional programs observed in mesenchymal subtypes, these findings suggest that developmental patterning of the gut wall emerges from a combination of early regional allocation and local differentiation programs.

Beyond lineage reconstruction, in utero nano-injection enables conditional gene manipulation in defined ENS populations. We show that Cre-dependent lentiviral cargo can be activated in differentiating enteric neurons or temporally controlled progenitor/glial populations. Proof-of-principle experiments with *Ascl1* demonstrated that the system can be used to test gene function in vivo during defined developmental windows. This approach complements emerging fetal atlases and disease genetics studies, which increasingly identify candidate regulators in transient embryonic cell states but often lack efficient in vivo validation strategies.

Several limitations should also be considered. Our results highlight a strong temporal component of lineage accessibility and commitment. Targeting at E7.5^Early^ enhanced labeling of ENS precursors but also increased cross-germ-layer clones, consistent with presence of epiblast cells. While residual epiblast labeling complicates clonal interpretation, it also provides a functional readout of when germ layers are consolidated in vivo. Tissue dissociation, single-cell capture, and barcode recovery also introduce dropouts that will inevitably reduce clonal reconstruction efficiency. Several general considerations apply to lentivirus approaches, including potential effects of integration site on cellular behavior and silencing of integrated transgenes (34). Conditional targeting efficiencies in the ENS were relatively modest. However, this mosaicism may be advantageous for studying cell-intrinsic mechanisms as ENS development depends on coordinated proliferation and long-range migration. Broad perturbation within the ENS may cause large secondary effects at the population level, whereas mosaic manipulation may reveal how genetically altered cells behave within an otherwise normal developing ENS.

Finally, this work has implications for understanding developmental origins of GI disease. Gut organogenesis relies on interdependent differentiation of diverse cell populations from all three germ layers, and subtle deviations in lineage allocation, maturation, or regional patterning may have lasting functional consequences (35). This is relevant not only to congenital disorders such as Hirschsprung disease and CIPO, but also to more common conditions in which early developmental alterations may predispose to later dysfunction. Some of these disorders may result from de novo mutations (36). It is conceivable that the impact of such mutations would be more predictable with a clearer understanding of refined cell lineages arising in postgastrula stages. We propose that this platform will provide a scalable strategy for mechanistic testing of candidate disease regulators, and modeling early cellular events that may contribute to GI disorders and enteric neuropathologies.

## Materials and Methods

Detailed materials and methods are provided in *SI Appendix*, *Supporting Information Text*. Briefly, high-titer lentiviral vectors (*SI Appendix*, Fig. S12 *A* and *B*) were delivered to mouse embryos by ultrasound-guided in utero nano-injection at E7.5 to enable lineage tracing and genetic manipulation. Embryonic tissues were analyzed by immunohistochemistry, confocal microscopy, and quantitative image analysis. Single-cell transcriptomic datasets were generated from flow cytometry-isolated cells (*SI Appendix*, Fig. S12 *C*–*H*) using the 10× Genomics platform and processed using standard quality-control, integration, and clustering pipelines. Cell annotations were validated through literature search (Dataset S1). CloneID extraction and clonal coupling analyses were performed to define lineage relationships among developing gut cell populations.

## Supplementary Material

Appendix 01 (PDF)

Dataset S01 (XLSX)

Dataset S02 (XLSX)

Dataset S03 (XLSX)

Dataset S04 (XLSX)

Dataset S05 (XLSX)

Dataset S06 (XLSX)

Dataset S07 (XLSX)

Dataset S08 (XLSX)

## Data Availability

Raw and processed single-cell RNA-sequencing data are available at Gene Expression Omnibus (GEO) database under the identifier GSE325733 (37). scRNA-seq data used in Fig. 1 can be found at ArrayExpress with the accession number E-MTAB-14817 (38). Code used to analyze data is summarized at https://github.com/UMarklundLab/Inutero_clonaltracing_E16Gut (39). Summary statistics of generated scRNA-seq dataset can be found in Dataset S2. Differentially expressed genes for clusters presented in the study are available in Datasets S3–S8. We adapted in situ hybridization images from the database Gene Paint (40) for *SI Appendix*, Fig. S4 *I*–*G* and the spatial transcriptomic reproductions in *SI Appendix*, Fig. S1*E* are adapted from the Mouse Organogenesis Spatiotemporal Transcriptomic Atlas (MOSTA) interactive viewer at https://db.cngb.org/stomics/mosta/ (13).
