# Supplementary material for: Resolving cell lineages and gene functions in the developing mouse gastrointestinal tract using in utero transduction
Source: Proc Natl Acad Sci U S A. 2026 Sep 9;123(37):e2614077123. doi: 10.1073/pnas.2614077123 (PMC13578780; doi:10.1073/pnas.2614077123)
Supplement: Supplementary file 1 [file pnas.2614077123.sapp.pdf]

PNAS

Supporting Information for

Resolving cell lineages and gene functions in the developing mouse gastrointestinal tract using *in utero* transduction

Ziwei Liu[1,‡], Krishnanand Padmanabhan[1,‡], Jingyan He[2,†], Katrin Hector[2], Bettina Semsch[2,3], Jia Sun[3], Viktoria Knoflach[1], Sarantis Giatrellis[2], Johan Lorentz[2], Khachatur Dallakyan[1], Christian Göritz[2], Emma Rachel Andersson[2,*], Ulrika Marklund[1,*]

[1] Department of Medical Biochemistry and Biophysics, Karolinska Institutet, Sweden
[2] Department of Cell and Molecular Biology, Karolinska Institutet, Sweden
[3] Comparative Medicine, Karolinska Institutet, Sweden
† Current Adress: Janelia Research Campus, Howard Hughes Medical Institute, Ashburn, USA
‡These two authors contributed equally
*These two authors share corresponding authorship

Corresponding author names: Ulrika Marklund and Emma Rachel Andersson
Email: Ulrika.Marklund@ki.se; Emma.Andersson@ki.se

This PDF file includes:

Supporting Information Text: Supplemental Material and Methods
Figures S1 to 10, including figure legends
Figure legends for Dataset S1 to S8
References for SI Appendix and Dataset S1

Other supporting materials for this manuscript include the following:

Dataset S1 to S8

Supporting Information Text

Supplemental Material and Methods:

Mice

The following transgenic and reporter mouse strains were used: Baf53b-Cre (JAX, #027826), Sox10-CreERT2 (SER26) (1), R26-EYFP (JAX #006148) and R26-tdTomato (Ai14; JAX #007908). C57/B6J and CD1 (Charles River Laboratories) wild-type (WT) mice were used. Animals were group-housed, with food and water *ad libitum,* under 12-h light-dark cycle conditions, 22°C ambient temperature and 50% humidity. Animal experiments were approved by the local ethics committee (Stockholm Norra djurförsöksetiska nämnd, Jordbruksverket; N5264/18, N6626-2019, N5237-2023, 8188-2017, 2987-2020 and 315-23).

Lentivirus constructs

Plasmid *LV-H2B-GFP* was a gift from Elaine Fuchs (Addgene #25999). *LV-EF1A-H2B-tdTomato-30N* was previously described (2, 3). *Lenti-DIO-EGFP* (SI Appendix, Fig. S12A) was based on the interim vector *pRRLSIN-cPPT-hPGK-EGFP-WPRE* (Gift from Didier Trono, Addgene #12252). *Lenti-DIO-EGFP-Ascl1* (SI Appendix, Fig. S12B) was constructed based on *pRRLSIN-cPPT-shortCAG-dlox-EGFP_2A(PTV)(rev)-dlox-WPRE* and the GeneArt product *mAscl1* (710bp). Both vectors were constructed, amplified, and characterized by the Viral Vector Facility (VVF) of the Neuroscience Center Zurich (ZNZ).

Plasmid preparation

Plasmid *LV-H2B-GFP* (Addgene plasmid #25999), *Lenti-DIO-EGFP, Lenti-DIO-EGFP-P2A-Ascl1* were transformed into bacteria, which were grown in 100mL Terrific Broth containing 100µg/mL Carbenicillin, on a horizontal shaker for 16-18 hours (h). Bacteria

were centrifuged to pellet at 4000 x g for 20 minutes (min) at 4°C. For DNA extraction PureLink™ or Macherry Nagel Endotoxin-Free Maxi Plasmid Purification Kit were used and concentrations measured by NanoDrop. All lentiviral constructs are third-generation lentiviral vectors.

Production of lentiviruses

High-titer vesicular stomatitis virus G (VSV-G)–pseudotyped lentiviral particles were produced either in-house or by a commercial facility (GEG TECH, France). For in house production, low passage (p4-p6) HEK 293 cell line with stable large T antigen expression (Lenti-XTM 293T cells (Clontech) or HEK293 FT cells (Thermofisher)) were thawed one week prior to virus production and grown at 37°C/5% CO_2 in growth medium (DMEM high glucose, GlutaMAX pyruvate, supplemented with 10% fetal bovine serum (FBS) and 1% penicillin/streptomycin (Pen/Strep). To sustain stable large T antigen expression, Geneticin (1%) was included in the growth medium during routine maintenance and propagation but was omitted during viral production and transduction procedures. Viral production was performed by transient transfection of the cells followed by cell culture for up to 60 h post-transfection. Calcium phosphate–based and polymer-based transfection reagents, including polyethyleneimine (PEI) and PolyJet (SignaGen laboratories), were used, both methods yielding high viral titers exceeding 1×10^9 transducing units (TU)/mL. At 12-16 h post-transfection, the growth medium with serum (Polyjet transfection protocol) or without serum (Calcium phosphate and PEI transfection protocol) was replaced with fresh growth media. Viral supernatant was collected every 24 h until 60 h post-transfection, with fresh medium replenished at each collection. Approximately 280 ml of pooled culture supernatant was concentrated to a final volume of <4 ml by multiple rounds of centrifugation through 100 kDa molecular weight cutoff centrifugal filter units (Millipore

Centricon 70 Plus) at 3,300 × g and 4°C, with individual centrifugation steps lasting 30–60 min.

The concentrated supernatant was transferred to ultracentrifuge tubes, and viral resuspension buffer (VRB; 20 mM Tris-HCl, pH 8.0, 250 mM NaCl, 10 mM $MgCl_2$, 5% sorbitol) was added to a final volume of 4 ml. The mixture was homogenized by vigorous pipetting. To form a sucrose cushion, 500 µl of 20% (w/v) sucrose in PBS was carefully layered at the bottom of each ultracentrifuge tube, creating a distinct phase beneath the viral suspension. Viral particles were pelleted by ultracentrifugation at 45,000 rpm for 1.5 h using an MLS-50 rotor. Following centrifugation, the supernatant was removed, and the viral pellet was resuspended in 20–30 µL of VRB or PBS. Aliquots were stored at −80°C.

Lentivirus titration

Lentiviral titers were determined by flow cytometry or quantitative (q)PCR. HEK293FT or NE4C cells were used as indicator cells. Cells were seeded in 6-well plates, and the cell number was determined after 12 h from one well. The remaining wells were transduced with serial dilutions of lentiviral supernatant in infection medium (DMEM high glucose, GlutaMAX pyruvate, growth medium supplemented with 20% FBS and 0.1 mg/mL polybrene). Plates were centrifuged at 1100 × g for 30 min at 37°C (spinoculation). The infection mixture was replaced with fresh growth medium, and cells were incubated at 37°C. 96 h after transduction; cells were trypsinized and analyzed by flow cytometry. Alternatively, GFP-positive cells were quantified by fluorescence microscopy. Infectious titers (infectious units per milliliter, IFU/mL) were calculated using the formula: IFU/mL = (% GFP-positive cells × number of cells at the time of transduction) / (volume of virus × dilution factor). For qPCR-based titration, viral RNA was isolated using the NucleoSpin RNA Virus kit (Macherey-Nagel), and lentiviral genome copies were quantified using the Lenti-X qPCR Titration Kit (Takara Bio) according to the manufacturer’s instructions.

Validation of DIO lentiviral constructs

Low-passage HEK293FT cells (P4–P6) were cultured at 37°C in 5% CO_2 and seeded at 5 × 10^4 cells per well in poly-L-lysine–coated 6-well plates upon reaching ~85% confluence. After 24 h, cells were transduced with lentiviral Cre-Puro particles (1 × 10^7 particles per well) in infection medium, followed by centrifugation at 1,100 × g for 30 min to enhance transduction efficiency. Medium was replaced post-centrifugation, and 48 h later, cells underwent puromycin selection (1 µg/mL) for 3 days. Surviving cells were expanded and cryopreserved. Stable Cre-expressing HEK293FT cells were reseeded under identical conditions and transduced with Lenti-DIO-EGFP or Lenti-DIO-EGFP-Ascl1 lentiviral particles (1 × 10^7 particles per well) using the same infection protocol. At 96 h post-transduction, cells were replated onto poly-L-lysine–coated glass coverslips in 24-well plates at 2 × 10^4 cells per well. The cells were fixed 24 h later in 4% paraformaldehyde (PFA) for 10 min at room temperature (RT) and permeabilized with 0.3% Triton X-100 for 5 min, followed by immunolabeling for ASCL1 and GFP using standard procedures. Briefly, cells were blocked with phosphate-buffered saline (PBS) containing 0.5% bovine serum albumin (BSA) for 1 h, followed by incubation with primary antibodies for 1 h at RT. Coverslips were washed three times with PBS and incubated with secondary antibodies for 1 h at room temperature. After three additional PBS washes, coverslips were mounted onto glass slides for imaging.

Ultrasound-guided *in utero* nano-injections

A detailed description of the procedure has been reported (4, 5). Briefly, pregnant females were anesthetized and placed in the supine position on a heated surgical platform maintained at 37 °C. Analgesia was administered prior to surgery. A minor laparotomy was performed to expose the uterus. At a given time, 2-3 embryos were gently exteriorized

through an elastic membrane positioned at the base of a modified Petri dish and immobilized using a columnar support of modeling compound. The dish was filled with PBS, and the amniotic cavities were visualized using a high-frequency ultrasound system (VEVO 2100, VisualSonics) with the probe submerged in PBS. A glass microcapillary needle (outer diameter, ~40 μm) attached to a nanoinjector (Nanoject III; Drummond Scientific) and loaded with viral vector was similarly lowered into the PBS and aligned with the ultrasound probe to permit visualization and targeting of the amniotic cavity. Amniotic cavities were injected with the maximal permissible volume, which was defined according to amniotic cavity size and mouse strain as described below. Embryos were gently returned to the maternal abdominal cavity. The muscular layer was closed with 6-0 Prolene sutures, and the skin was closed using EZ clips.

Comments on embryonic stage and injection parameters

High-resolution ultrasound imaging of embryos from CD1 and C57BL/6 mice enabled identification of all corresponding Theiler stages (TS) spanning the E7.0–8.5 developmental window, as defined by eMouseAtlas Project (SI Appendix, Fig. S3A-C). We considered Theiler stages TS11A–C to represent the optimal E7.5 window for ultrasound-guided *in utero* injections (SI Appendix, Fig. S3A, B), while injection at E8 or later failed to efficiently target gut and ENS (SI Appendix, Fig. 3C, D). Strain-specific differences were observed, with CD1 embryos displaying larger amniotic cavities and overall embryonic structures, facilitating *in utero* nano-injection. Embryos at TS11A-C, tolerated volumes of lentiviral particles ranged from 30 to 207 nL, consistent with our earlier studies (4, 5). The more robust CD1 embryos were used for lineage tracing experiments and assessment of labeling efficiency. All Cre driver lines for ENS cell type–specific genetic perturbations were maintained on the C57BL/6 background.

Tamoxifen administration

Plug-mated females received tamoxifen (0.1 mg/g body weight; Sigma) dissolved in corn oil/ethanol (9:1) by intraperitoneal injection at E16.0-E16.5. Embryos were collected 26 or 55 hours after tamoxifen administration.

Tissue preparation and immunohistochemistry

E13.5 embryos were decapitated and fixed in 4% PFA in PBS for 4-5 hours at 4 °C. Intestines from E14.5-E18.5 were dissected out and fixed in 4% PFA in PBS at 4 °C for 1.5-2 hours. Samples were washed in PBS three times and dehydrated in 30% sucrose in PBS at 4 °C overnight. Intestines were cut into 1 cm long pieces, representing different regions and embedded for transverse analysis in OCT freezing medium and stored at -80 °C. Samples were sectioned at 12 μm and stored at -20 °C.

Frozen tissue sections were air-dried at RT for 30 min, rinsed with PBS and incubated in blocking solution containing purified donkey anti-mouse Fab (Jackson Laboratories; 715-007-003) diluted 1:50 in PBS for 2 hours at RT. After rinsing with PBS, the sections were blocked with 2% normal donkey serum (NDS, Jackson) and 0.1% Triton X-100 (Sigma) in PBS for 2 hours, and incubated with primary antibodies diluted in the same solution overnight at 4°C. They were then washed three times with PBS (10 minutes each) and incubated with secondary antibodies at RT for 1 hour. Tissue was washed three times with PBS (10 minutes each) and mounted in DAKO mounting medium (Agilent) containing DAPI (4',6-diamidino-2-phenylindole).

Primary antibodies used: chicken anti-GFP (1:1000; Abcam AB13970), sheep anti-GFP (1:500; Bio-Rad 4745-1051), mouse anti-NeuN (1:200; Millipore MAB377), goat anti-SOX2 (1:200; Santa Cruz SC-17230), mouse anti-HuC/D (1:300; Molecular Probes A21271), rabbit anti-HuC/D (1:300; Abcam ab184267), human anti-HuC/D (1:20 000; ANNA-1 gift from David Linden and Vanda Lennon, Mayo Clinic, Rochester, US), goat

anti-SOX10 (1:100-500; R&D AF2864), rabbit anti-SOX10 (1:300; Clone #EPR4007, Abcam ab155279), rabbit anti-S100 (1:1000; DAKO Z0311), rabbit anti-αSMA (1:500; Abcam ab5694), rat anti-E-cadherin (1:200; Biolegend 147301), rabbit anti-ASCL1 (1:500; Abcam ab211327), rat anti-Ki67 (1:500; ThermoFisher Scientific 740008T), rabbit anti-cleaved Caspase3 (5A1E) (1:500; Cell Signaling Technology 9664), and goat anti-NeuroD (1:100; Santa Cruz Biotechnology sc-1086 G-20).

Secondary antibodies used: donkey anti-chicken 488 (1:400; Jackson ImmunoResearch 703-545-155), donkey anti-sheep 488 (1:400; ThermoFisher Scientific A11015), donkey anti-rabbit 555 (1:1000; ThermoFisher Scientific A31572), goat anti-mouse IgG2b 555 (1:1000; ThermoFisher Scientific A21147), donkey anti-goat 647 (1:250; ThermoFisher Scientific A21447), donkey anti-Rat 647 (1:250; ThermoFisher Scientific A48272), donkey anti-human Cy5 (1:250; Jackson Immuno Research 709-165-149) and DAPI (1:1000; Sigma D9542), and donkey anti-Rat 555 (1:1000; ThermoFisher Scientific A78945).

Cell counting and statistical analysis

Immunofluorescence staining was assessed and images acquired using the Zeiss LSM confocal microscopes in the series LSM710 - LSM980 Airy. Animals from different litters were used for repeat experiments to avoid confounding. Quantifications were performed on confocal images, using 'cell counter' plugin in ImageJ or manually. For DRG quantifications 8 sections were used per embryo, 134μm apart and spanning from thoracic to sacral levels. Data were analyzed using GraphPad Prism 9 and presented as mean ± SD. Statistical significance was assessed using unpaired two-tailed t-test for the data presented in Figure 6H-J, L, M and O, with Welch's correction applied for L, M and O(high).

Embryonic tissue preparation for single-cell RNA sequencing

CD1 embryos injected with LV-EF1A-H2B-tdTomato-30N were harvested at E16.5. Stomach and 1/3 of the distal small intestine were harvested from the E7.5 experiment (one embryo), while the stomach, 2/3 of the small intestine, and the entire large intestine were harvested from the E7.5^{Early} and E7.5^{Early2} experiments (one embryo each). Tissue was kept in cold DMEM/F12 medium (Invitrogen) on ice during dissection. The mesentery was removed, the intestines were cut into 2–5mm^2 pieces and put into a digestion solution (0.75 mg/ml Liberase TH (research grade, Roche), 0.1 mg/ml DNase I (Sigma), 4 U/ml dispase (Corning®, 354235) for stomach, 3 U/ml dispase for small and large intestine, in DMEM/F12) at 37 °C for 15 min for the stomach or 12 min for the small and large intestine with shaking every 3 min. The digestion mixture was replaced with DMEM/F12 medium containing 2% BSA (ThermoFisher) and 5 mM EDTA (Invitrogen). The cells were then manually triturated using three fire-polished Pasteur pipettes with decreasing bore diameters (from ~70% to 10% of the original bore diameter) that were previously coated with 1% BSA solution at RT. The single-cell suspension was filtered through a DMEM/F12-equilibrated 30-μm cell strainer (Miltenyi Biotec) and centrifuged at 200 x g for 10 min at 4°C. Cells were resuspended in DPBS (Gibco) containing 1% BSA and then incubated with DRAQ7 Dye (1:200) (Biostatus) for 5 min before sorting. Tomato+ DRAQ7- cells were sorted into a 1.5mL Eppendorf tube coated with 1% BSA using a BD Influx (v7) Cell Sorter or a BD FACSAria Fusion equipped with a 100 μm nozzle and were collected in ice-cold DPBS containing 0.04% BSA. Gating was performed using BD FACS software v.1.0.0.650 or BD FACSDiva (v9.0.1) (see examples in SI Appendix, Fig. S12 C-H). After sorting, samples were centrifuged at 200 x g for 8 min at 4 °C, and supernatants were carefully discarded.

Single cell RNA-sequencing

cDNA libraries were prepared according to the manufacturer's instructions, using 10x Chromium Next GEM Single Cell 3' Reagent Kits Version 3.1 Dual Index (10x Genomics, CA, USA). Cell suspensions were adjusted to 500–1,000 cells per µl and added to 10x Chromium RT mix to achieve target cell recovery of between 3,000–10,000 cells per reaction. The libraries were sequenced on Illumina NovaSeq 6000 or XPlus platforms using Novaseq S4 reagent kit; 300 cycles. The reads were trimmed to 28-10-10-90 and demultiplexed on the Data Delivery System at NGI Sweden. The datasets were named according to estimated stage of injection (i) and analysis (a): iE7.5-aE16.5, iE7.5^{Early}-aE16.5 and iE7.5^{Early2}-aE16.5.

Analysis of single cell RNA-sequencing datasets

Analysis of E9.5 and E10.5 datasets

Single cell RNA-sequencing datasets from Lentivirus-injected 9.5 and E10.5 embryos (2) were re-analyzed using Scanpy (v1.11.4). The neural crest cell cluster was subsetted from the full dataset and reprocessed to identify subpopulations. Specifically, counts were renormalized, highly variable genes (HVGs) were identified within the subset, and principal component analysis (PCA), neighbourhood graph construction, Uniform Manifold Approximation and Projection (UMAP) embedding, and Leiden clustering were performed Subcluster identities were assigned based on the expression of top marker genes identified by differential gene expression analysis using the Wilcoxon rank-sum test (sc.tl.rank_genes_groups) as implemented in Scanpy.

Single-cell RNA sequencing preprocessing for E16.5 embryos

A custom reference was generated by combining the mouse reference genome GRCm38 (mm10, version 2020-A) genome and an additional sequence representing tdTomato-N transgene, in which the barcode region is marked with 'N' 30-bp barcode wildcard characters using 10x Genomics CellRanger v5.0.(2, 3) Single-cell sequence FASTQ files were aligned to this customized reference using CellRanger-v7.1.0 and v9.0.1. Raw single-cell count matrices for all samples were imported using Seurat version 5 in a renv-managed R environment (R v4.5.0).

Ambient RNA assessment and correction

Ambient RNA was evaluated from paired raw and filtered count matrices using SoupX(v1.6.2). Empty droplets were defined as barcodes with <100 total UMIs (unique molecular identifiers), and background expression profiles were computed across these droplets. Contamination fractions were estimated using SoupX (SoupChannel, setClusters, autoEstCont) with quick clustering (scran::quickCluster) to define groups for estimation. For the iE7.5^{Early}-aE16.5 and iE7.5^{Early2}-aE16.5 datasets, ambient RNA correction was applied, SoupX-corrected count matrices were generated by first building a Seurat object from CellRanger filtered counts, normalisation and scaling, PCA, a k-nearest neighbor (k-NN) graph, Louvain clustering at resolution 0.5, and using these clusters as SoupX groups; corrected counts were generated with adjustCounts and exported in 10x Genomics v3 format using DropletUtils::write10xCounts.

Doublet detection and removal

Doublets were detected per sample using DoubletFinder (v2.0.6). Low-quality cells expressing <2000 genes, <2000 UMIs, or > 10% mitochondrial genes were excluded before downstream analysis. Potential doublets expressing >8000 genes and >50000 gene counts were filtered out. For each sample, preliminary preprocessing was performed

(NormalizeData, variable feature selection with 3000 features, ScaleData, PCA, UMAP, a k-NN graph, Louvain clustering at resolution 0.8) to obtain group labels used for homotypic doublet estimation. DoubletFinder parameters were selected by sweeping pK (paramSweep/summarizeSweep/find.pK) and choosing the value maximizing the BC metric; the expected number of doublets was set from a sample-specific expected doublet rate and adjusted by the estimated homotypic proportion (modelHomotypic). DoubletFinder was run using PCs 1:30 with pN = 0.25, and cells classified as doublets were excluded from downstream analyses.

Normalization, dimensionality reduction, integration, and clustering

Normalization and variance stabilization was performed using SCTransform (v0.4.3), regressing out the mitochondrial percentage. The top 3000 HVGs, excluding the sex-associated genes (*Xist*, *Gm13305*, *Tsix*, *Gm8730, Eif2s3y*, *Ddx3y*, *Uty, Kdm5d*) were used for PCA. Cells were clustered using a k-NN graph and Leiden clustering, followed by visualization using UMAP. Batch correction was performed using the first 30-50PCs for Harmony Integration. The resulting Harmony embeddings were used to construct a k-NN graph, followed by Leiden clustering and UMAP visualization.

Post hoc quality control was performed to remove residual poor-quality cells that formed distinct clusters characterized by high mitochondrial percentages, as well as potential doublets identified by high total UMIs high total gene number, and co-expression of markers genes from distinct lineages. The remaining cells were reprocessed through a final round of normalization, dimensionality reduction, Harmony integration, and clustering. The final datasets comprised 5,356 cells from the iE7.5-aE16.5 dataset, 21,749 cells from the iE7.5^{Early}-aE16.5 dataset, and 15,430 cells from the iE7.5^{Early2}-aE16.5 dataset. Each cell was assigned a cell-cycle state using the CellCycleScoring function. The ENS cell population from the iE7.5-aE16.5 dataset was subsequently subsetted and

processed with an additional round of normalization, dimensionality reduction, and clustering.

For comparative analyses, Harmony was applied to integrate the iE7.5–aE16.5 and iE7.5^{Early}–aE16.5 datasets using the first 30 PCs following SCTransform normalization while regressing out both mitochondrial percentage and the difference between the S phase and G2M phase cell cycle scores prior to PCA. Harmony was also used to generate a fully integrated dataset comprising all three samples using 50 PCs following SCTransform normalization while regressing out mitochondrial percentage prior to PCA.

Cell-type annotation

Cell clusters were manually annotated based on assessing the top differentially expressed genes identified from the Wilcoxon rank-sum test (FindAllMarkers) implemented in Seurat, together with the expression of canonical marker genes for gut cell types. Cell type annotations were further benchmarked against predictions generated by an automated annotation tool - Single Cell Signature Scorer for Annotation (SCSA) (6) with hyperparameters LFC = 1.5 and p-value = 0.05. Dataset S1 summarises the cell type annotations of clusters with positive and negative marker genes and supporting references. Differentially Expressed Gene lists can be found in Dataset S3-8.

CloneID construction

We extracted the cell IDs from all cells of the same embryo that passed the quality control and exported the cell IDs as a TSV file for barcode extraction and CloneID constructionimplemented in the TREX pipeline (3). TREX uses reads from filtered cells, based on imported cell IDs, to align to the tdTomato-N transgene, and barcodes were recovered from alignment spanning the N region. Overrepresented barcodes identified from lentiviral barcode library sequencing and characterization (2) were excluded from

CloneID construction. Barcodes retrieved with shorter than 7 nucleotides were also removed. Reads with identical barcode sequence; UMI and cell ID were collapsed into a consensus sequence. To correct sequencing errorse, barcode sequences weree single-linkage clustered using a Hamming distance of 5. Cells were assigned the same CloneID when they shared both the same number of barcodes and identical barcode sequences, corresponding to a Jaccard index threshold of 1.0, which represents the most stringent criterion for clone assignment.

Clonal composition and clonal coupling analysis

CloneIDs were mapped onto the Seurat metadata of each dataset using matched cell IDs to annotate cells as either 'with CloneID' or 'without CloneID', and where applicable, assigned a corresponding CloneID (clone_nr). Clone size was defined as the number of cells sharing the same CloneID. Clones were categorized as singleton (one cell), single-cell-type multicellular (≥2 cells belonging to one annotated cell type), or multi-cell-type multicellular (≥2 cells belonging to more than one annotated cell type). For clonal composition analyses, cells were annotated to fibroblasts, pericytes, interstitial cells of Cajal (ICC), smooth muscle cells, myofibroblasts, mesothelial cells, endothelial cells, simple epithelium, foregut basal epithelium, ENS, and immune cells. Clone sharing across annotated cell types and gut regions was summarized using UpSet plots and chord diagrams. Regionally dispersed clones were determined by clones with cells present in more than one gut region (stomach, small intestine, or large intestine). To quantify over- or under-representation of cell types sharing clones, clonal coupling scores were calculated as previously described (7, 8). Observed clone sharing was compared to randomized data, yielding a z-score between each pair of annotated cell types. The resulting clonal coupling z-scores represent how many standard deviations the observed clone sharing between each cell type pair is away from the mean of randomized data.

Positive z-score values indicate higher than expected clone sharing and negative z-score values indicate lower than expected clone sharing. Analyses were performed using 1,000-3000 random shuffles for multiple times.

To identify annotated cell types with similar clonal coupling patterns, Pearson correlations of z-scores were computed by which cell types are grouped by similarities in clonal coupling z-score profiles in complete lineage hierarchical clustering using Euclidean distance. Positive correlation values indicate that two cell types exhibit similar patterns of clonal coupling with other cell types, whereas negative correlation values indicate dissimilar clonal coupling patterns. Summary of experimental datasets used for E16.5 clonal relationships analysis can be found in Dataset S2.

To test the likelihood that cell-type abundance explains multi-cell-type clone differences between E1(iE7.5-aE16.5) and E2(iE7.5-aE16.5^{Early}), we performed Monte Carlo composition-matching. This included the removal of 3,783 colonic cells (as colon was not captured in E1) and thereafter repeated down-sampling to match the exact numbers of each major cell type of E1. We performed 1,000 stratified down-samplings of E2 to E1 cell-type counts and accounted for the presence of multi cell-type multicellular clones involving the ENS or immune cells. Down-sampled E2 showed mean shared-clone counts of 16.9 clones (ENS) and 6.3 clones (Immune) vs. 0 in E1 (both $p \approx 0.001$; 0/1,000 iterations matching E1). As E2 consisted of generally larger clones than E1, we additionally addressed the consequence of removing large clones prior to the down-sampling procedure. Removing E2 clones >200 cells had little effect (ENS mean 16.4 clones, $p \approx 0.001$; Immune mean 6.0 clones, $p \approx 0.001$). When E2 was restricted to clones no larger than the maximum clone size of E1 (31 cells), sharing was still prominent albeit reduced (ENS mean 4.7 clones, 1/1,000 iterations matching E1, $p \approx 0.002$; Immune mean 3.0 clones, 0/1,000 iterations matching E1, $p \approx 0.001$). These analyses indicate that the presence of multi-cell-type clones (cross-germ layer clones) involving the ENS and

immune cells observed in E2 cannot be explained by differences in cell-type abundance alone. The Monte Carlo p-values were calculated as (k+1)/(N+1), where k is the number of down-sampling iterations producing zero multi-cell-type clones, matching the observation in E1, and N=1000 iterations. The Phipson-Smyth correction was used to avoid obtaining p values of exactly zero as we cannot conclude that the true probability is zero given a finite number of iterations.

Data, Materials, and Software Availability

Raw and processed single cell RNA-sequencing data are available at Gene Expression Omnibus (GEO) database under the identifier GSE325733. scRNA-seq data used in Fig. 1 can be found at ArrayExpress with the accession number E-MTAB-14817. Code used to analyze data are summarized at https://github.com/liuziweisteph/Inutero_clonaltracing_E16Gut. Summary statistics of generated scRNA-seq dataset can be found in Dataset S2. We adapted *in situ* hybridization images from the database Gene Paint (9) in SI Appendix, Fig. S1E S4I-G and the spatial transcriptomic reproductions in SI Appendix Fig. S1E are adapted from the Mouse Organogenesis Spatiotemporal Transcriptomic Atlas (MOSTA) interactive viewer at https://db.cngb.org/stomics/mosta/ (10).

Supplementary Figures:

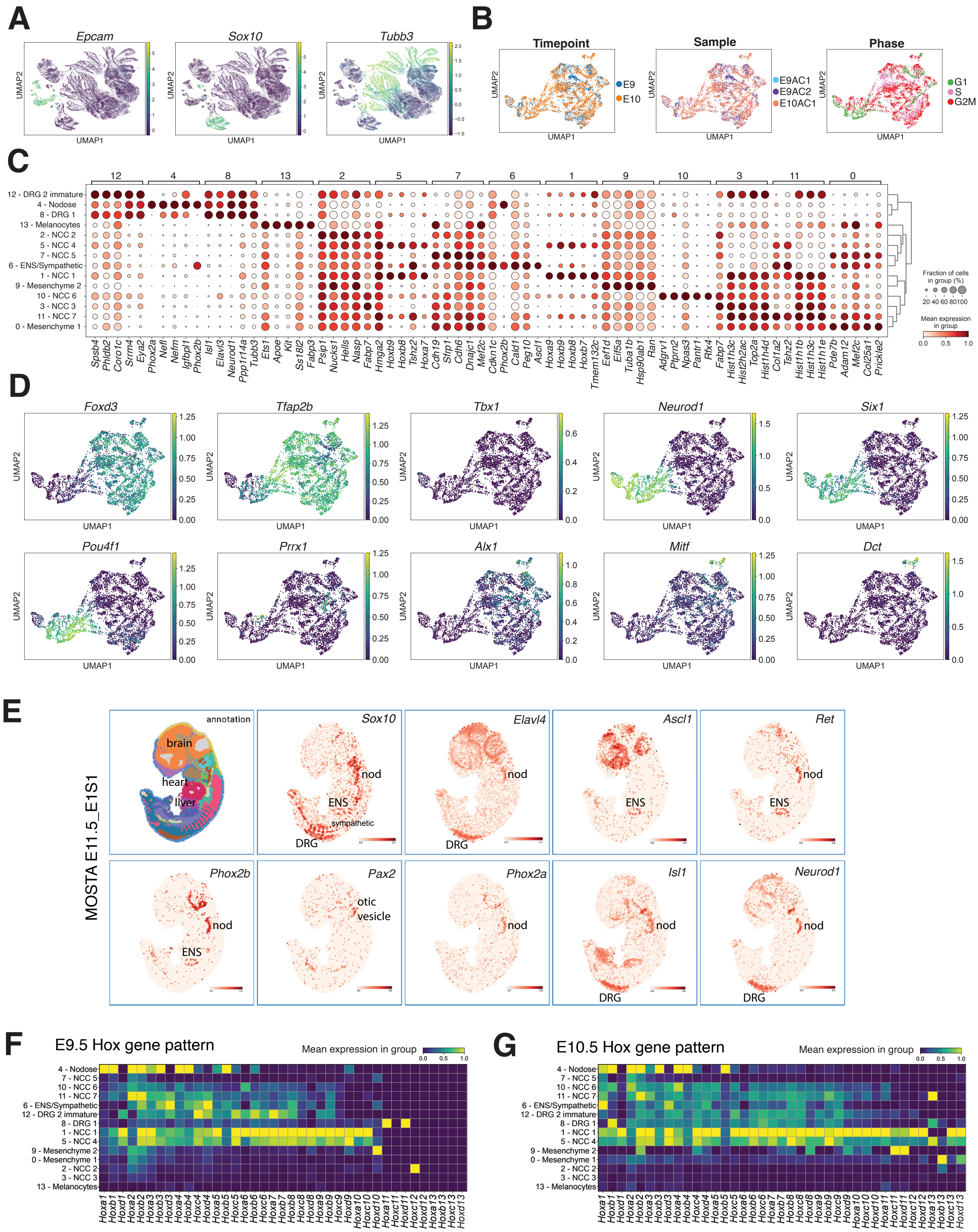
A
Epcam
Sox10
Tubb3
UMAP1
UMAP2
B
Timepoint
E9
E10
Sample
E9AC1
E9AC2
E10AC1
Phase
G1
S
G2M
C
12 - DRG 2 immature
4 - Nodose
8 - DRG 1
13 - Melanocytes
2 - NCC 2
5 - NCC 4
7 - NCC 5
6 - ENS/Sympathetic
1 - NCC 1
9 - Mesenchyme 2
10 - NCC 6
3 - NCC 3
11 - NCC 7
0 - Mesenchyme 1
Spsb4 Phldb2 Coro1c Srrm4 Eya2 Phox2a Nefl Nefm Igfbpl1 Phox2b Isl1 Elavl3 Neurod1 Ppp1r14a Tubb3 Ets1 Apoe Kit Ss18l2 Fabp3 Psip1 Nucks1 Hells Nasp Fabp7 Hmga2 Hoxb9 Hoxb8 Tshz2 Hoxa7 Cdh19 Sfrp1 Cdh6 Dnajc1 Mef2c Cdkn1c Phox2b Cad1 Peg10 Ascl1 Hoxa9 Hoxb9 Hoxb8 Hoxb7 Tmem132c Eef1d Eif5a Tuba1b Hsp90ab1 Ran Adgrv1 Ptprn2 Npas3 Pantr1 Rfx4 Fabp7 Hist1h3c Hist2h2ac Top2a Hist1h4d Col1a2 Tshz2 Hist1h1b Hist1h3c Hist1h1e Pde7b Adam12 Mef2c Col25a1 Prickle2
Fraction of cells in group (%)
20 40 60 80 100
Mean expression in group
0.0 0.5 1.0
D
Foxd3
Tfap2b
Tbx1
Neurod1
Six1
Pou4f1
Prrx1
Alx1
Mitf
Dct
E
MOSTA E11.5_E1S1
annotation
brain
heart
liver
Sox10
nod
ENS
sympathetic
DRG
Elavl4
Ascl1
Ret
Phox2b
Pax2
otic vesicle
Phox2a
Isl1
Neurod1
F
E9.5 Hox gene pattern
Mean expression in group
G
E10.5 Hox gene pattern
Hoxa1 Hoxb1 Hoxd1 Hoxa2 Hoxb2 Hoxa3 Hoxb3 Hoxd3 Hoxa4 Hoxb4 Hoxd4 Hoxa5 Hoxb5 Hoxc5 Hoxa6 Hoxb6 Hoxc6 Hoxa7 Hoxb7 Hoxb8 Hoxc8 Hoxd8 Hoxa9 Hoxb9 Hoxc9 Hoxd9 Hoxa10 Hoxc10 Hoxd10 Hoxa11 Hoxc11 Hoxd11 Hoxc12 Hoxd12 Hoxa13 Hoxc13 Hoxd13

Fig. S1. Supportive data related to Figure 1 on neural crest/placode derived cells at E9.5-E10.5 (A) Feature plots of representative marker genes for epithelial cells (*Epcam*+), neural crest (*Sox10*+) and neurons (*Tubb3*+). (**B**) UMAP plot of annotated neural crest/placode-derived cells, colored by developmental timepoint, sample identity and cell-cycle phase. (**C**) Dot plot displaying expression of the top five differentially expressed genes for each cluster. Dot size represents the fraction of cells expressing the gene and color indicates normalized mean expression. (**D**) Feature plots of selected marker genes for neural crest (*Foxd3*+, *Tfap2b*+) and distinct neural crest/placode lineages, including nodose, dorsal root ganglia (DRG), cranial mesenchyme, and melanocyte populations. (**E**) Spatial gene expression pattern of E11.5_E1S1, an E11.5 sagittal embryo subjected to Stereo-Seq. Images were adapted from the Mouse Organogenesis Spatiotemporal Transcriptomic Atlas (MOSTA) interactive viewer via STOmicsDB (Dataset ID: STDS0000058) available at https://db.cngb.org/stomics/mosta/. Combinatorial gene patterns identify nodose ganglion (*Sox10, Elavl4, Ret, Phox2b, Phox2a, Isl1, Neurod1*) below the otic vesicle (*Pax2*), ENS (*Sox10, Elavl4, Ret, Ascl1, Phox2b*), and DRG (*Sox10, Elavl4, Isl1, Neurod1*). Sympathetic ganglia are most noticeable with *Sox10*. Note that stomach mesenchyme expresses Isl1. (**F-G**) Heatmaps showing expression of Hox genes across annotated neural crest/placode-derived cell populations at E9.5 (F) and E10.5 (G)

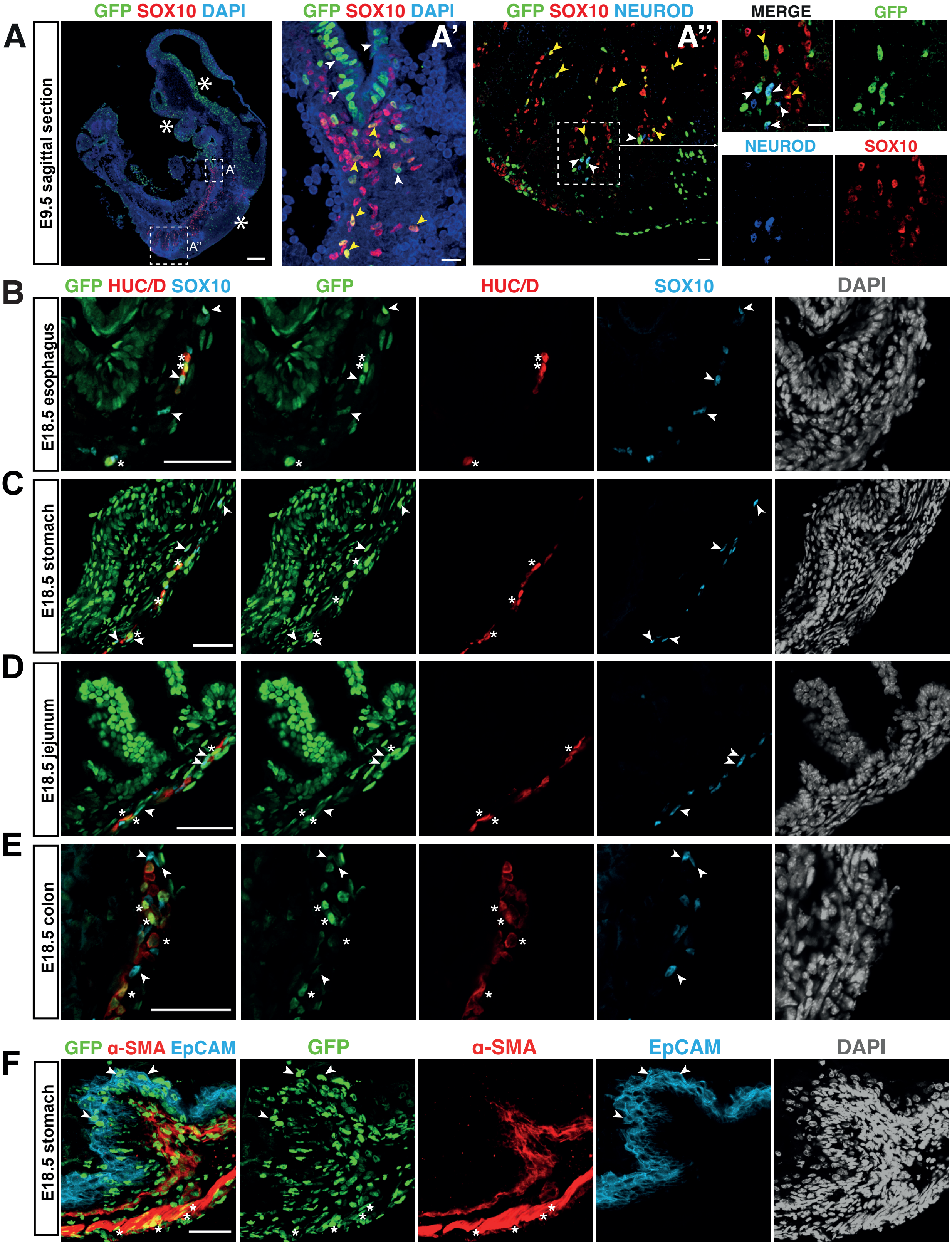
A
GFP SOX10 DAPI
E9.5 sagittal section
A'
A''
GFP SOX10 DAPI
A'
GFP SOX10 NEUROD
A''
MERGE
GFP
NEUROD
SOX10
B
GFP HUC/D SOX10
GFP
HUC/D
SOX10
DAPI
E18.5 esophagus
C
E18.5 stomach
D
E18.5 jejunum
E
E18.5 colon
F
GFP α-SMA EpCAM
GFP
α-SMA
EpCAM
DAPI
E18.5 stomach

Fig. S2. ***In utero*** **transduction of LV-H2B-GFP at E7.5 results in widespread GFP expression throughout the ENS and gut wall across different gut regions.** (**A**) Immunofluorescence image of a sagittal section through an entire transduced E9.5 embryo. Scale bar, 200 µm. (**A'**) Higher magnification view of the foregut showing GFP expression in both ENS (SOX10+, yellow arrowheads) and non-ENS (SOX10-, white arrowheads) cells. Scale bar, 20µm. Stars indicate transduction sites in the brain, spinal cord and branchial arches. Dashed boxes indicate areas magnified in A' and A''. (**A''**) Higher magnification view of the trunk region showing GFP expression in emerging DRG neurons (NEUROD+, white arrowheads) and DRG/Sympathetic progenitors (SOX10+, yellow arrowheads). Scale bars, 20µm (**B-E**) Representative immunofluorescence images showing GFP expression in HuC/D^{+} neurons (asterisks) and SOX10^{+} ENS progenitors/glia (arrowheads) in different gut regions at E18.5. (**F**) Representative immunofluorescence image of the stomach collected at E18.5 showing GFP expression in α-SMA^{+} smooth muscle cells (asterisks) and EpCAM^{+} epithelial cells (arrowheads). Scale bars, 50 µm.

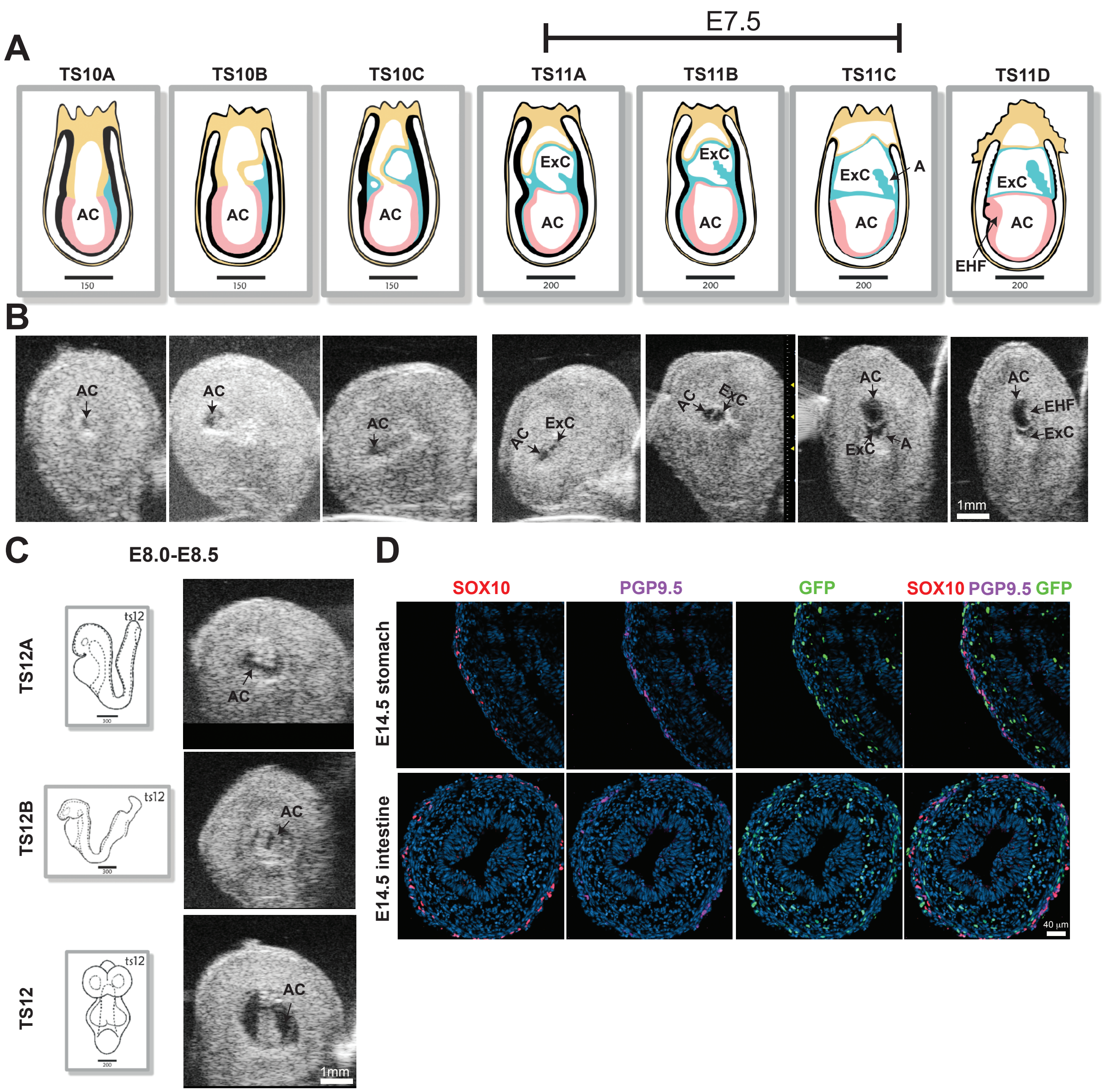

Fig. S3. Schematic drawings and representative ultrasound images of mouse Theiler stages.
(A) Schematic drawings of E7.0-E7.75 mouse embryos showing different embryonic structures. TS11a-11c represent approximately the E7.5 stage. Images adapted from the eMouseAtlas Project (www.emouseatlas.org). **(B)** Corresponding ultrasound images of E7.0-E7.75 mouse embryos with structures labelled. Scale bar: 1mm. **(C)** Schematic drawings from the eMouseAtlas Project of TS12A-C, representing approximately E8.0-E8.25. Representative ultrasound images of E8.0-E8.5 mouse embryos. Scale bar: 1mm. **(D)** Representative confocal images of E14.5 stomach and intestine transduced with LV-H2B-GFP in E8.0-E8.5 embryos showing only sparse labelling of mesenchyme, and no epithelia nor ENS (PGP9.5+ neurons; SOX10+ glia/progenitors). Scale bar: 40μm.
AC: amniotic cavity; ExC: extracoelomic cavity; A: allantois and EHF: early head fold

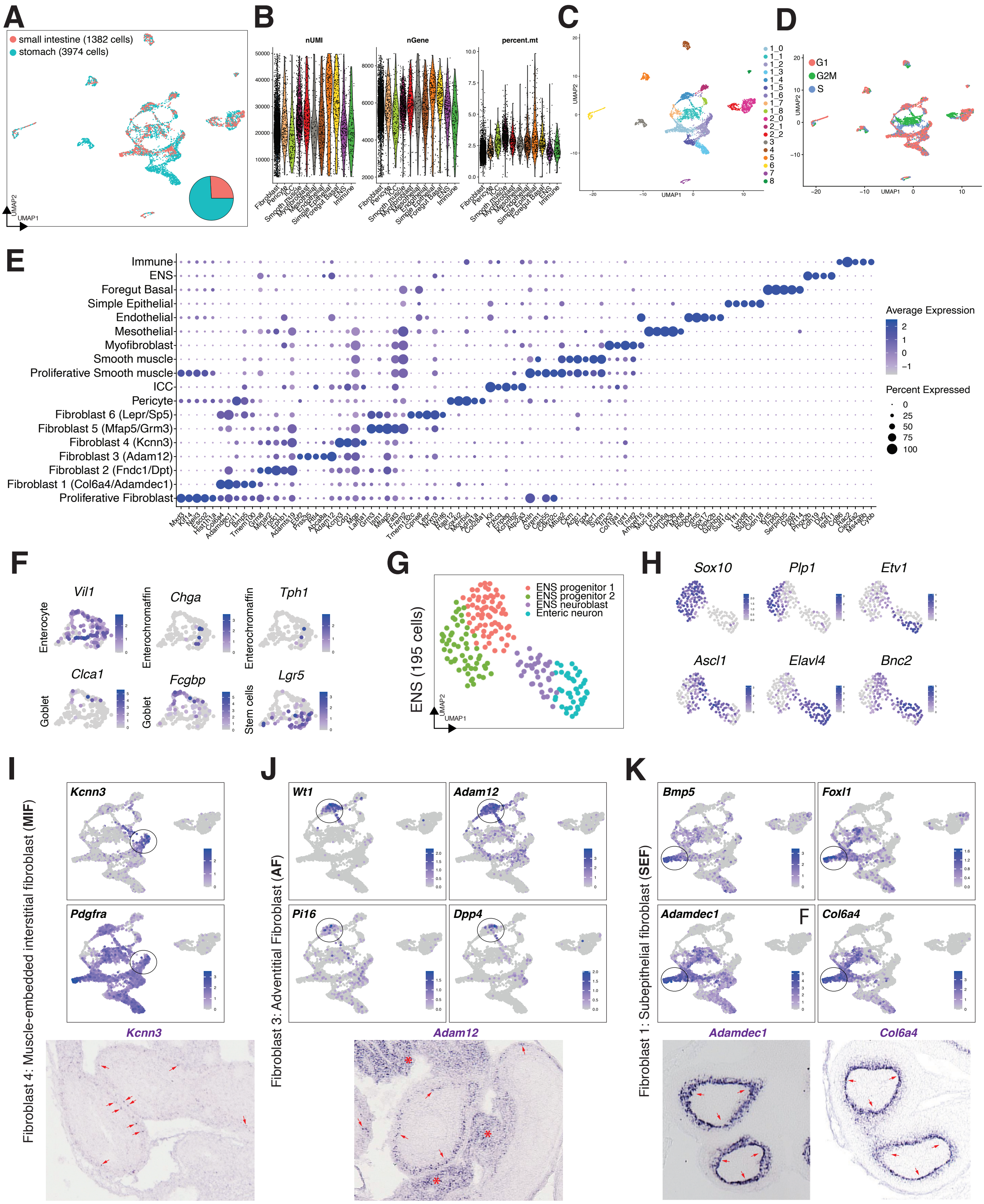
A
small intestine (1382 cells)
stomach (3974 cells)
UMAP2
UMAP1
B
nUMI
nGene
percent.mt
C
D
G1
G2M
S
E
Immune
ENS
Foregut Basal
Simple Epithelial
Endothelial
Mesothelial
Myofibroblast
Smooth muscle
Proliferative Smooth muscle
ICC
Pericyte
Fibroblast 6 (Lepr/Sp5)
Fibroblast 5 (Mfap5/Grm3)
Fibroblast 4 (Kcnn3)
Fibroblast 3 (Adam12)
Fibroblast 2 (Fndc1/Dpt)
Fibroblast 1 (Col6a4/Adamdec1)
Proliferative Fibroblast
Average Expression
Percent Expressed
F
Enterocyte
Vil1
Enterochromaffin
Chga
Enterochromaffin
Tph1
Goblet
Clca1
Goblet
Fcgbp
Stem cells
Lgr5
G
ENS (195 cells)
ENS progenitor 1
ENS progenitor 2
ENS neuroblast
Enteric neuron
H
Sox10
Plp1
Etv1
Ascl1
Elavl4
Bnc2
I
Fibroblast 4: Muscle-embedded interstitial fibroblast (MIF)
Kcnn3
Pdgfra
Kcnn3
J
Fibroblast 3: Adventitial Fibroblast (AF)
Wt1
Adam12
Pi16
Dpp4
Adam12
K
Fibroblast 1: Subepithelial fibroblast (SEF)
Bmp5
Foxl1
Adamdec1
Col6a4
Adamdec1
Col6a4

Fig S4. Quality control and marker gene analysis of gut cell types in the iE7.5-aE16.5 dataset. (A) UMAP plot of gut cells colored by tissue of origin. Pie chart indicates the proportion of cells from each region. **(B)** Violin plots showing per-cell quality control metrics for each major cell type after filtering, including total reads (nUMI), number of unique genes detected (nGene), and percentage of mitochondrial transcripts (percent.mt). **(C)** UMAP plot of cells colored by unsupervised cluster assignment. **(D)** UMAP plot of cells colored by cell-cycle phase. **(E)** Dot plot displaying expression of the top five differentially expressed genes for each refined cell type. Dot size represents the fraction of cells expressing the gene, and color indicates relative mean expression. (**F**) Feature plots displaying marker gene expression of epithelial cell types. (**G**) UMAP plot of reprocessed ENS cells. (**H**) Feature plots showing expression of selected markers for ENS states and branches. **(I)** Feature plots showing markers of Muscle-embedded Interstitial Fibroblasts (MIF) in Fibroblast 4. **(J)** Feature plots showing expression of genes associated with Adventitial Fibroblasts (AF) in Fibroblast 3. **(K)** Feature plots showing expression of genes associated with Subepithelial Fibroblasts (SEFs) in Fibroblast 1. Insets in (**I-K**) display *in situ* hybridization images of representative markers for the three fibroblast clusters from E14.5 mouse sagittal sections adapted from the database Gene Paint. *Kcnn3* is expressed in the muscle walls (arrows), in agreement with the annotation as MIFs. *Adam12* is expressed outer wall (arrows) consistent with a linkage to serosal cells (AF) and is additionally expressed in mesentery (stars). *Adamdec1* and *Col6a4* are confined to the subepithelial space (arrows) in agreement with a SEF identity.

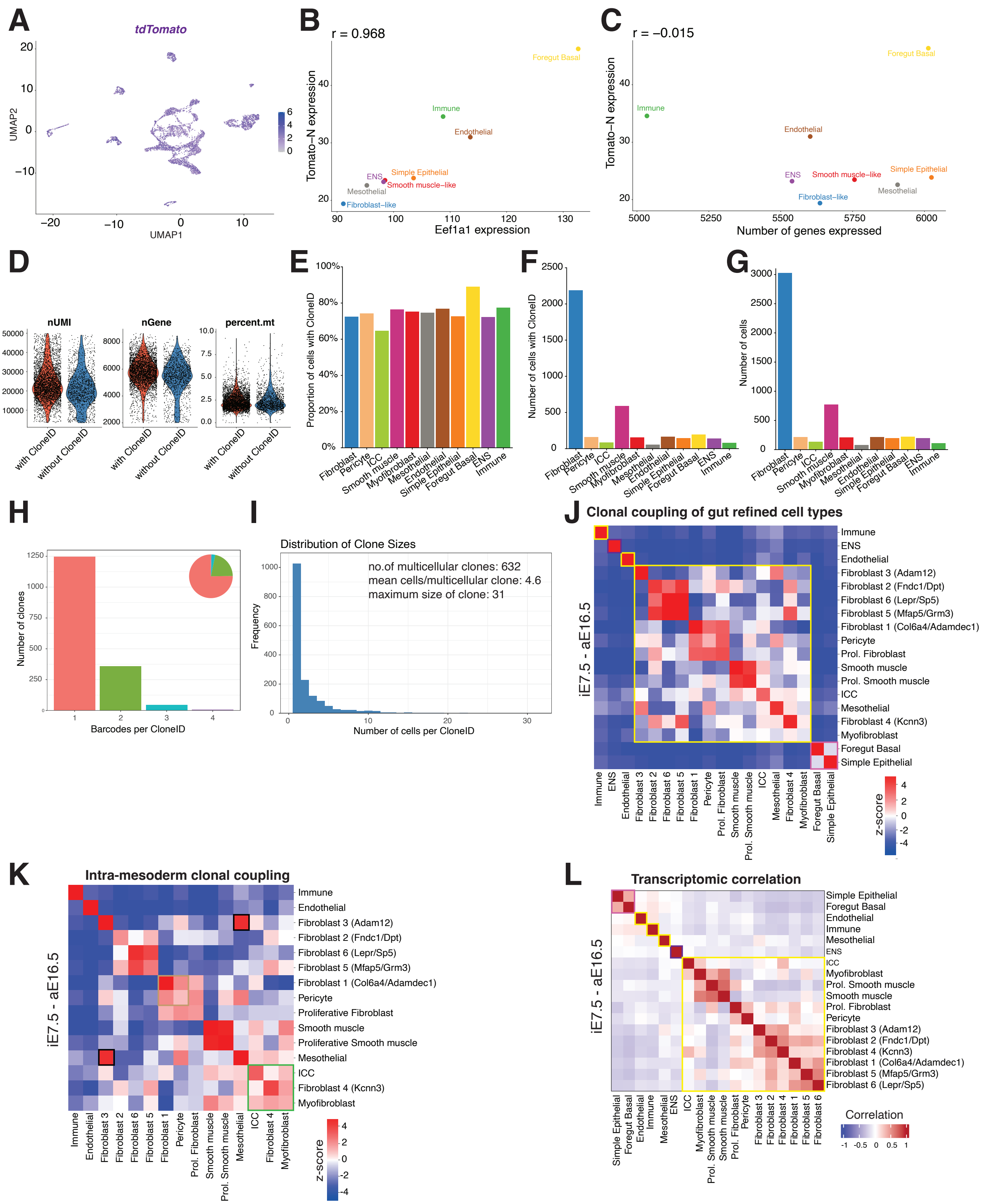
A
tdTomato
UMAP2
UMAP1
B
r = 0.968
Tomato–N expression
Eef1a1 expression
Foregut Basal
Immune
Endothelial
ENS
Simple Epithelial
Smooth muscle–like
Mesothelial
Fibroblast–like
C
r = −0.015
Tomato–N expression
Number of genes expressed
D
nUMI
nGene
percent.mt
with CloneID
without CloneID
E
Proportion of cells with CloneID
F
Number of cells with CloneID
G
Number of cells
Fibroblast
Pericyte
ICC
Smooth muscle
Myofibroblast
Mesothelial
Endothelial
Simple Epithelial
Foregut Basal
ENS
Immune
H
Number of clones
Barcodes per CloneID
I
Distribution of Clone Sizes
no.of multicellular clones: 632
mean cells/multicellular clone: 4.6
maximum size of clone: 31
Frequency
Number of cells per CloneID
J
Clonal coupling of gut refined cell types
iE7.5 - aE16.5
Immune
ENS
Endothelial
Fibroblast 3 (Adam12)
Fibroblast 2 (Fndc1/Dpt)
Fibroblast 6 (Lepr/Sp5)
Fibroblast 5 (Mfap5/Grm3)
Fibroblast 1 (Col6a4/Adamdec1)
Pericyte
Prol. Fibroblast
Smooth muscle
Prol. Smooth muscle
ICC
Mesothelial
Fibroblast 4 (Kcnn3)
Myofibroblast
Foregut Basal
Simple Epithelial
z-score
K
Intra-mesoderm clonal coupling
iE7.5 - aE16.5
Immune
Endothelial
Fibroblast 3 (Adam12)
Fibroblast 2 (Fndc1/Dpt)
Fibroblast 6 (Lepr/Sp5)
Fibroblast 5 (Mfap5/Grm3)
Fibroblast 1 (Col6a4/Adamdec1)
Pericyte
Proliferative Fibroblast
Smooth muscle
Proliferative Smooth muscle
Mesothelial
ICC
Fibroblast 4 (Kcnn3)
Myofibroblast
z-score
L
Transcriptomic correlation
iE7.5 - aE16.5
Simple Epithelial
Foregut Basal
Endothelial
Immune
Mesothelial
ENS
ICC
Myofibroblast
Prol. Smooth muscle
Smooth muscle
Prol. Fibroblast
Pericyte
Fibroblast 3 (Adam12)
Fibroblast 2 (Fndc1/Dpt)
Fibroblast 4 (Kcnn3)
Fibroblast 1 (Col6a4/Adamdec1)
Fibroblast 5 (Mfap5/Grm3)
Fibroblast 6 (Lepr/Sp5)
Correlation

Fig. S5. CloneID assessment and clonal coupling analysis in the iE7.5-aE16.5 dataset. (A) Feature plot displaying *tdTomato* expression. **(B)** Scatter plot showing a high Pearson correlation between *Eef1a1* expression and *tdTomato* expression across major gut cell types, indicating that the synthetic Eef1a1 promoter recapitulates endogenous *Eef1a1* expression patterns. **(C)** Scatter plot showing a low Pearson correlation between *tdTomato* expression and average number of genes detected per cell across major gut cell type, excluding library complexity as a confounder. **(D)** Violin plots showing per-cell quality control metrics for cells with and without assigned CloneIDs. **(E)** Bar graph displaying the proportion of cells with CloneIDs per cell type. **(F)** Bar graph showing the number of cells with CloneIDs per cell type. **(G)** Bar graph showing the total number of cells in each cell type. (H) Bar graph and pie chart illustrating the distribution of the number of unique barcodes per CloneID. **(I)** Bar graph showing distribution of the number of cells per CloneID, with summary statistics. **(J, K)** Heatmaps of clonal coupling z-scores, defined as the deviation in the number of shared CloneIDs between pairs of cell types relative to randomized data. Analyses were performed using all clones (**J**) or clones restricted to mesoderm-derived cells (**K**) related to Figure 3F and 3G. **(L)** Heatmap showing transcriptomic correlations between refined cell types based on gene expression profiles. Colored boxes denote germ-layer origin of the cell type (**J**, **L**; pink: endoderm; purple: ectoderm; yellow: mesoderm) or lineage coupled mesenchymal populations (**K**; green: Fibroblast 4 with myofibroblasts, ICC and smooth muscle cells; black: Fibroblast 3 with mesothelial cells; brown: Fibroblast 1 with pericytes).

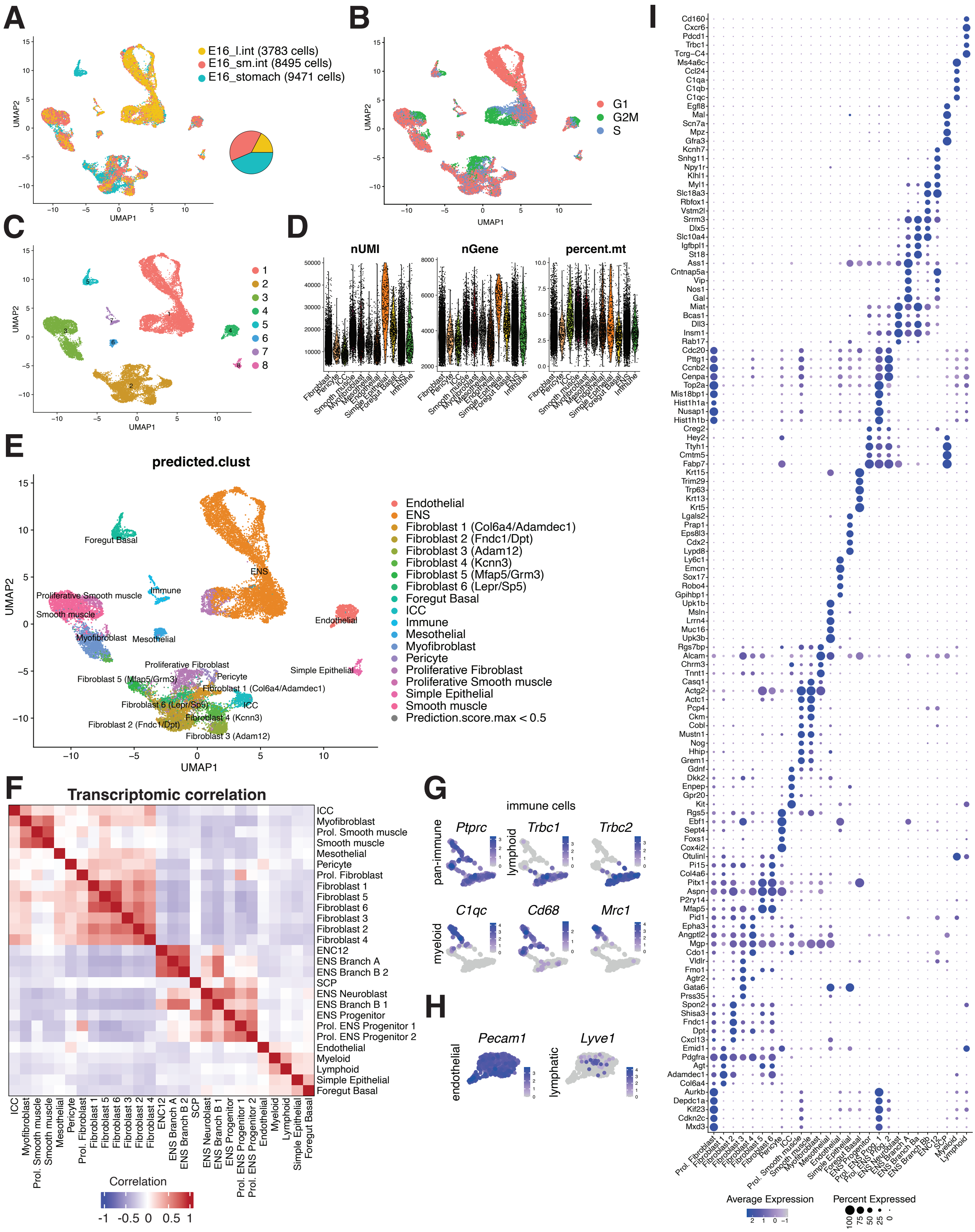

A
E16_l.int (3783 cells)
E16_sm.int (8495 cells)
E16_stomach (9471 cells)
UMAP1
UMAP2
B
G1
G2M
S
C
1
2
3
4
5
6
7
8
D
nUMI
nGene
percent.mt
E
predicted.clust
Foregut Basal
ENS
Proliferative Smooth muscle
Immune
Smooth muscle
Endothelial
Myofibroblast
Mesothelial
Proliferative Fibroblast
Simple Epithelial
Fibroblast 5 (Mfap5/Grm3)
Pericyte
Fibroblast 1 (Col6a4/Adamdec1)
Fibroblast 6 (Lepr/Sp5)
ICC
Fibroblast 4 (Kcnn3)
Fibroblast 2 (Fndc1/Dpt)
Fibroblast 3 (Adam12)
Endothelial
ENS
Fibroblast 1 (Col6a4/Adamdec1)
Fibroblast 2 (Fndc1/Dpt)
Fibroblast 3 (Adam12)
Fibroblast 4 (Kcnn3)
Fibroblast 5 (Mfap5/Grm3)
Fibroblast 6 (Lepr/Sp5)
Foregut Basal
ICC
Immune
Mesothelial
Myofibroblast
Pericyte
Proliferative Fibroblast
Proliferative Smooth muscle
Simple Epithelial
Smooth muscle
Prediction.score.max < 0.5
F
Transcriptomic correlation
ICC
Myofibroblast
Prol. Smooth muscle
Smooth muscle
Mesothelial
Pericyte
Prol. Fibroblast
Fibroblast 1
Fibroblast 5
Fibroblast 6
Fibroblast 3
Fibroblast 2
Fibroblast 4
ENC12
ENS Branch A
ENS Branch B 2
SCP
ENS Neuroblast
ENS Branch B 1
ENS Progenitor
Prol. ENS Progenitor 1
Prol. ENS Progenitor 2
Endothelial
Myeloid
Lymphoid
Simple Epithelial
Foregut Basal
Correlation
-1 -0.5 0 0.5 1
G
immune cells
pan-immune
Ptprc
lymphoid
Trbc1
Trbc2
myeloid
C1qc
Cd68
Mrc1
H
endothelial
Pecam1
lymphatic
Lyve1
I
Average Expression
Percent Expressed

Fig. S6. Quality control and marker gene analysis of gut cell types in the iE7.5^{Early}-aE16.5 dataset. (A) UMAP plot of cells colored by sample origin (stomach, small intestine, large intestine). Pie chart indicates the proportion of cells contributed by each gut region. **(B)** UMAP plot of cells colored by cell-cycle phase. **(C)** UMAP plot of cells colored by unsupervised cluster assignment. **(D)** Violin plots showing per-cell quality control metrics for each major cell type after filtering, including total reads (nUMI), number of unique genes detected (nGene), and percentage of mitochondrial transcripts (percent.mt). **(E)** UMAP plot showing predicted cell-type annotations of the iE7.5^{Early}-aE16.5 dataset generated by label transfer using the iE7.5-aE16.5 dataset as a reference. **(F)** Heatmap showing transcriptomic correlations between refined cell types based on gene expression profiles. **(G)** Feature plots displaying gene expression of pan-immune (Ptprc), lymphoid (*Trbc1, Trbc2*) and myeloid (*Cd68, C1qc, Mrc1*) markers. **(H)** Feature plots displaying gene expression of pan-endothelial (*Pecam1*) and lymphatic (*Lyve1*) markers **(I)** Dot plot showing expression of the top five differentially expressed genes for each refined cell types. Dot size represents the percentage of cells expressing the gene, and color indicates relative mean expression.

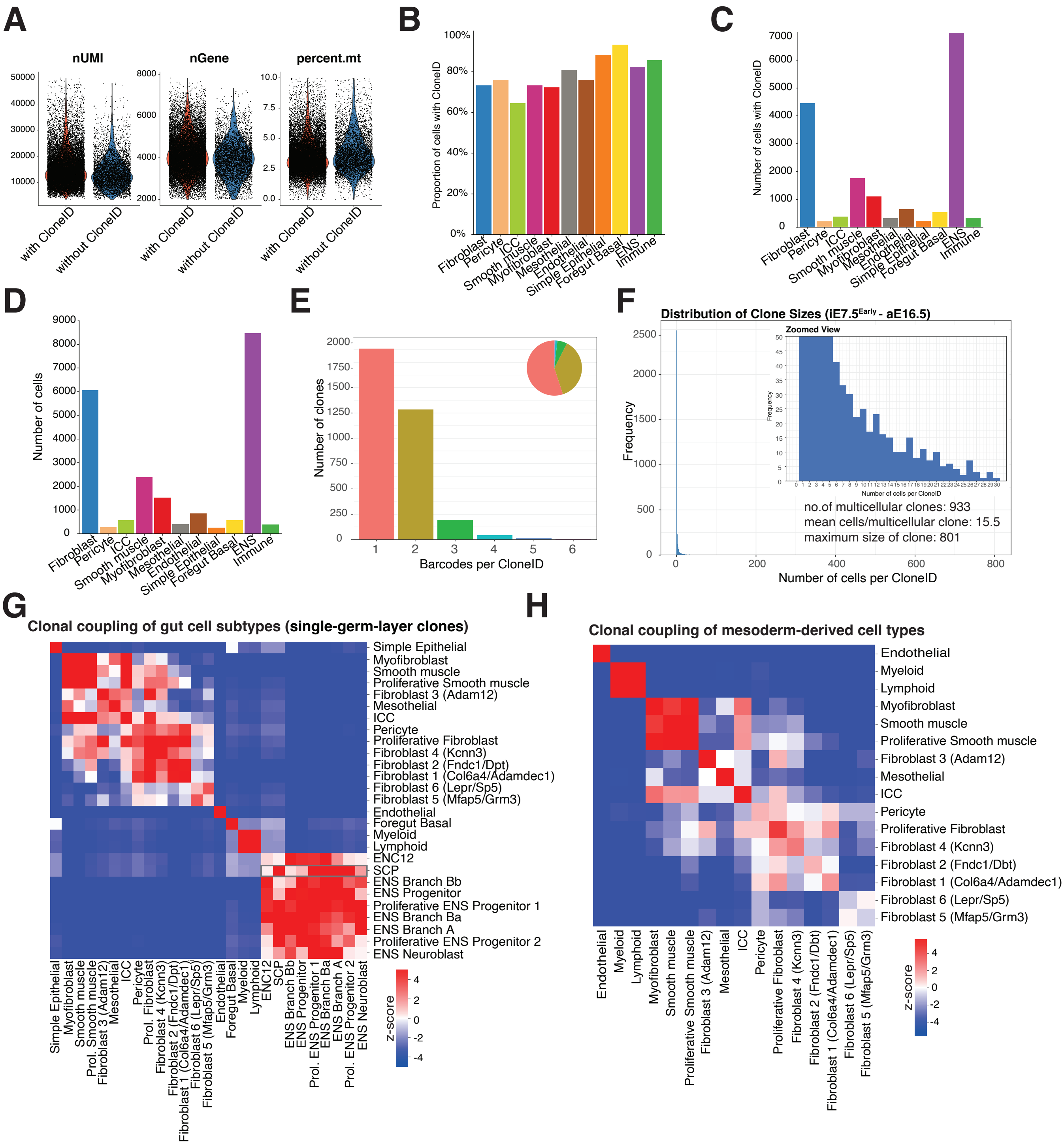
A
nUMI
nGene
percent.mt
with CloneID
without CloneID
B
Proportion of cells with CloneID
Fibroblast
Pericyte
ICC
Smooth muscle
Myofibroblast
Mesothelial
Endothelial
Simple Epithelial
Foregut Basal
ENS
Immune
C
Number of cells with CloneID
D
Number of cells
E
Number of clones
Barcodes per CloneID
F
Distribution of Clone Sizes (iE7.5Early - aE16.5)
Zoomed View
Frequency
no.of multicellular clones: 933
mean cells/multicellular clone: 15.5
maximum size of clone: 801
Number of cells per CloneID
G
Clonal coupling of gut cell subtypes (single-germ-layer clones)
Simple Epithelial
Myofibroblast
Smooth muscle
Proliferative Smooth muscle
Fibroblast 3 (Adam12)
Mesothelial
ICC
Pericyte
Proliferative Fibroblast
Fibroblast 4 (Kcnn3)
Fibroblast 2 (Fndc1/Dpt)
Fibroblast 1 (Col6a4/Adamdec1)
Fibroblast 6 (Lepr/Sp5)
Fibroblast 5 (Mfap5/Grm3)
Endothelial
Foregut Basal
Myeloid
Lymphoid
ENC12
SCP
ENS Branch Bb
ENS Progenitor
Proliferative ENS Progenitor 1
ENS Branch Ba
ENS Branch A
Proliferative ENS Progenitor 2
ENS Neuroblast
z-score
H
Clonal coupling of mesoderm-derived cell types
Endothelial
Myeloid
Lymphoid
Myofibroblast
Smooth muscle
Proliferative Smooth muscle
Fibroblast 3 (Adam12)
Mesothelial
ICC
Pericyte
Proliferative Fibroblast
Fibroblast 4 (Kcnn3)
Fibroblast 2 (Fndc1/Dbt)
Fibroblast 1 (Col6a4/Adamdec1)
Fibroblast 6 (Lepr/Sp5)
Fibroblast 5 (Mfap5/Grm3)
z-score

Fig. S7. CloneID assessment and clonal coupling analysis in the iE7.5^{Early}-aE16.5 dataset. **(A)** Violin plots showing per-cell quality control metrics for cells with and without CloneIDs. **(B)** Bar graph displaying the proportion of cells with CloneIDs in each cell type. **(C)** Bar graph showing the number of cells with CloneIDs in each cell type. **(D)** Bar graph showing the number of cells in each cell type. **(E)** Bar graph and pie chart illustrating the distribution of the number of unique barcodes per CloneID. **(F)** Bar graph showing the distribution of the number of cells per CloneID, with summary statistics. **(G,H)** Heatmaps of clonal coupling z-scores, defined as the deviation in the number of shared CloneIDs between pairs of cell types relative to randomized data. Analyses were performed using all single-germ-layer clones (**G**) or single-germ-layer clones with only mesoderm-derived cells (**H**), related to Figure 4H, 4I. Gray box in (**G**) highlights clonal coupling between SCP and all ENS states, but relatively lower coupling to the two most mature neuron clusters (ENC12 and ENS Branch Bb) in agreement with the later arrival of SCPs to the gut compared with vagal neural crest.

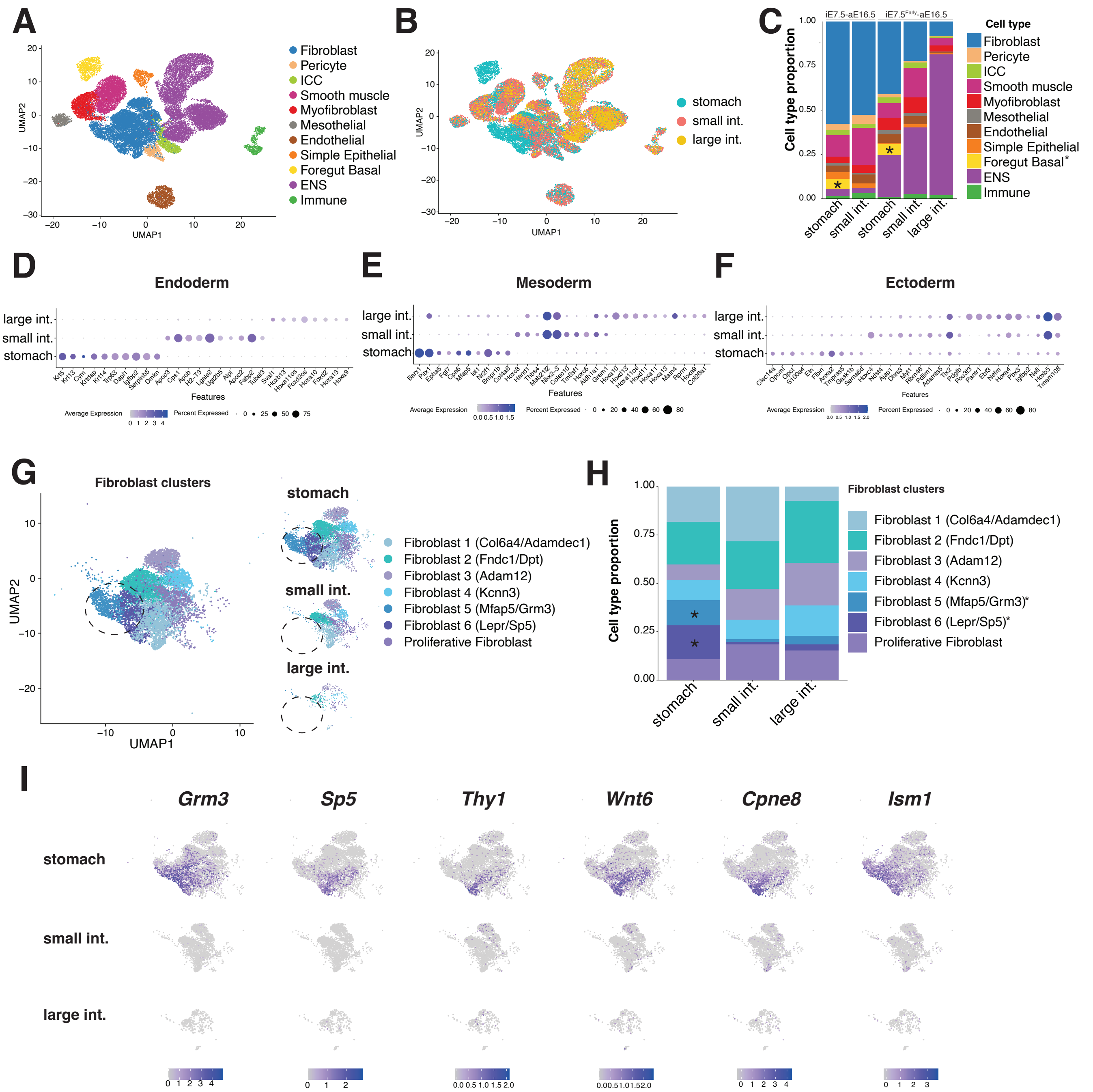
A
UMAP1
UMAP2
Fibroblast
Pericyte
ICC
Smooth muscle
Myofibroblast
Mesothelial
Endothelial
Simple Epithelial
Foregut Basal
ENS
Immune
B
UMAP1
UMAP2
stomach
small int.
large int.
C
Cell type proportion
iE7.5-aE16.5
iE7.5^Early-aE16.5
stomach
small int.
stomach
small int.
large int.
Cell type
Fibroblast
Pericyte
ICC
Smooth muscle
Myofibroblast
Mesothelial
Endothelial
Simple Epithelial
Foregut Basal*
ENS
Immune
D
Endoderm
large int.
small int.
stomach
Features
Average Expression
Percent Expressed
E
Mesoderm
large int.
small int.
stomach
Features
Average Expression
Percent Expressed
F
Ectoderm
large int.
small int.
stomach
Features
Average Expression
Percent Expressed
G
Fibroblast clusters
UMAP1
UMAP2
stomach
small int.
large int.
Fibroblast 1 (Col6a4/Adamdec1)
Fibroblast 2 (Fndc1/Dpt)
Fibroblast 3 (Adam12)
Fibroblast 4 (Kcnn3)
Fibroblast 5 (Mfap5/Grm3)
Fibroblast 6 (Lepr/Sp5)
Proliferative Fibroblast
H
Cell type proportion
stomach
small int.
large int.
Fibroblast clusters
Fibroblast 1 (Col6a4/Adamdec1)
Fibroblast 2 (Fndc1/Dpt)
Fibroblast 3 (Adam12)
Fibroblast 4 (Kcnn3)
Fibroblast 5 (Mfap5/Grm3)*
Fibroblast 6 (Lepr/Sp5)*
Proliferative Fibroblast
I
Grm3
Sp5
Thy1
Wnt6
Cpne8
Ism1
stomach
small int.
large int.

Fig. S8. Regional distribution of gut cell types and gene expression patterns. (A) UMAP plot of cells from the integrated iE7.5-aE16.5 and iE7.5^{Early}-aE16.5 datasets, showing overlap of major cell types across datasets. **(B)** UMAP plot of cells colored by tissue of origin. **(C)** Stacked bar plots showing the proportion of major cell types across gut regions and samples. Asterisks indicate Foregut Basal cells, which are detected exclusively in stomach samples. **(D–F)** Dot plots showing the top 10 differentially expressed genes of cells derived from each germ layer: endoderm (**D**), mesoderm (**E**), and ectoderm (**F**), across gut regions. Dot size represents the percentage of cells expressing each gene, and color indicates average expression level. **(G)** UMAP plot showing fibroblast subclusters across gut regions. Encircled area indicates fibroblast 5/6 that are predominantly isolated from the stomach. **(H)** Stacked bar plot showing the proportion of fibroblast subclusters across gut regions. Asterisks indicate fibroblast 5/6 enrichment in the stomach. **(I)** Feature plots showing regional expression patterns of genes enriched in Fibroblast 5/6 across gut regions. *Gm3* and *Sp5* showed stomach-specific expression, whereas Fibroblast 6 expresses *Wnt6*, *Cpne8* and *Ism1* supporting a fibroblast subpopulation with high signaling capacity, consistent with a crypt/submucosal niche. int.= intestine.

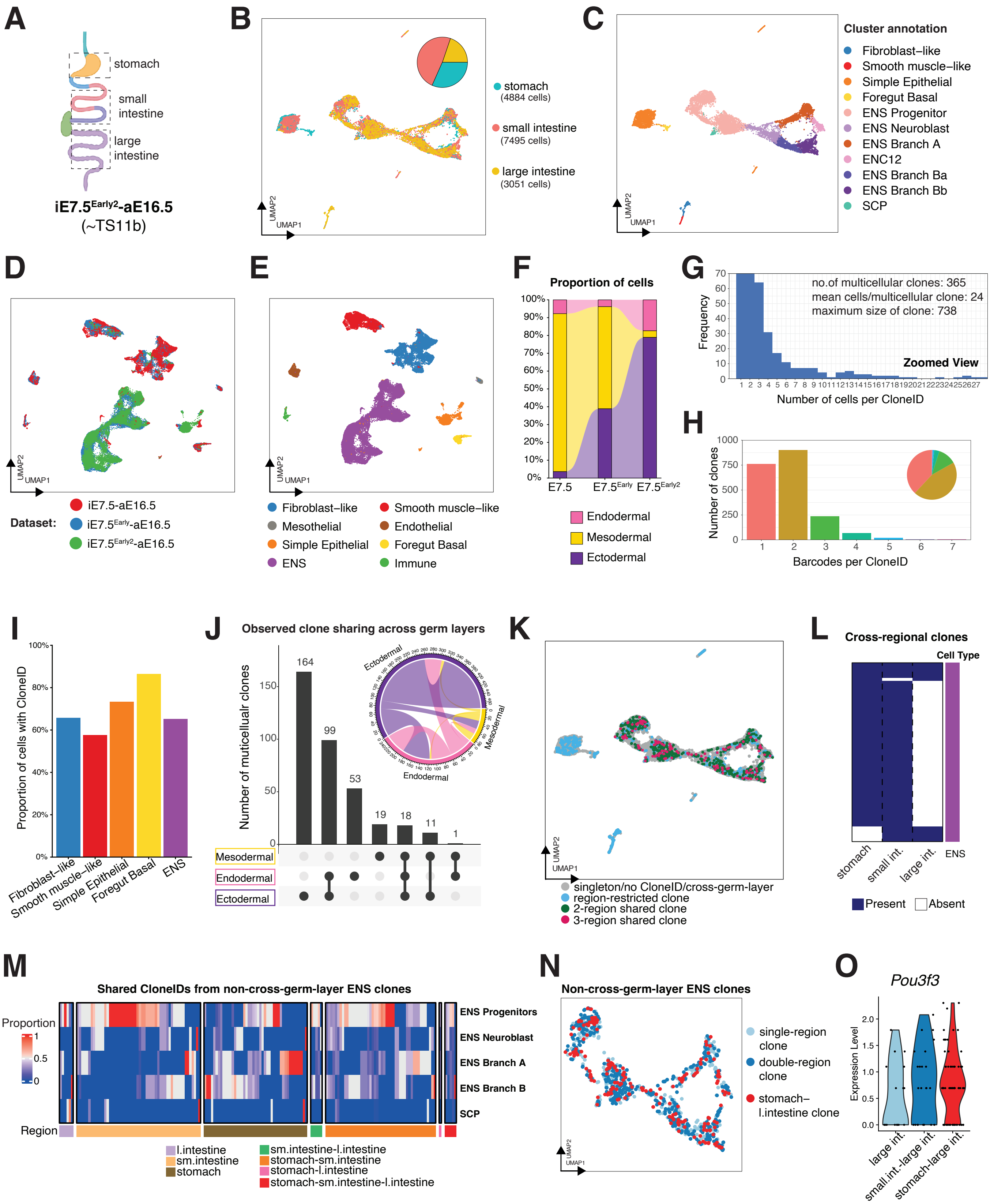
A
stomach
small intestine
large intestine
iE7.5Early2-aE16.5
(~TS11b)
B
stomach (4884 cells)
small intestine (7495 cells)
large intestine (3051 cells)
UMAP1
UMAP2
C
Cluster annotation
Fibroblast–like
Smooth muscle–like
Simple Epithelial
Foregut Basal
ENS Progenitor
ENS Neuroblast
ENS Branch A
ENC12
ENS Branch Ba
ENS Branch Bb
SCP
D
Dataset:
iE7.5-aE16.5
iE7.5Early-aE16.5
iE7.5Early2-aE16.5
E
Fibroblast–like
Smooth muscle–like
Mesothelial
Endothelial
Simple Epithelial
Foregut Basal
ENS
Immune
F
Proportion of cells
E7.5
E7.5Early
E7.5Early2
Endodermal
Mesodermal
Ectodermal
G
no.of multicellular clones: 365
mean cells/multicellular clone: 24
maximum size of clone: 738
Zoomed View
Frequency
Number of cells per CloneID
H
Number of clones
Barcodes per CloneID
I
Proportion of cells with CloneID
Fibroblast–like
Smooth muscle–like
Simple Epithelial
Foregut Basal
ENS
J
Observed clone sharing across germ layers
Number of muticellualr clones
164
99
53
19
18
11
1
Ectodermal
Mesodermal
Endodermal
K
singleton/no CloneID/cross-germ-layer
region-restricted clone
2-region shared clone
3-region shared clone
L
Cross-regional clones
Cell Type
stomach
small int.
large int.
ENS
Present
Absent
M
Shared CloneIDs from non-cross-germ-layer ENS clones
Proportion
ENS Progenitors
ENS Neuroblast
ENS Branch A
ENS Branch B
SCP
Region
l.intestine
sm.intestine
stomach
sm.intestine-l.intestine
stomach-sm.intestine
stomach-l.intestine
stomach-sm.intestine-l.intestine
N
Non-cross-germ-layer ENS clones
single-region clone
double-region clone
stomach–l.intestine clone
O
Pou3f3
Expression Level
large int.
small int.-large int.
stomach-large int.

Fig. S9. Validation of regional clonal coupling analysis using a third barcoded dataset (iE7.5^{Early2}-aE16.5). (A) Schematic drawing indicating the regions collected to generate the iE7.5^{Early2}-aE16.5 dataset. (**B**) UMAP plot showing cells colored by sample origin in the iE7.5^{Early2}-aE16.5 dataset. Pie chart indicates the proportions of cells contributed by each gut region: stomach (4,884 cells), small intestine (7,495 cells), and large intestine (3,051 cells). (**C**) UMAP plot showing major cell population clusters and ENS state clusters. (**D**) UMAP plot colored by datasets integrated by Harmony. Note that the three datasets merge well. (**E**) UMAP plot showing major cell populations in the integrated data from all three datasets. All three datasets contribute to all clusters, except the immune clusters, which is lacking from dataset 3. **(F)** Alluvial plot showing proportions of cells derived from each germ layer comparing datasets. Note that the iE7.5^{Early2}-aE16.5 dataset has the highest proportions of ectoderm- and endoderm-derived cells representing ENS and epithelial populations. **(G)** Bar graph showing the distribution of cells per CloneID, with summary statistics for the iE7.5^{Early2}-aE16.5 dataset. **(H)** Bar graph and pie chart showing the distribution of barcodes per CloneID in the iE7.5^{Early2}-aE16.5 dataset. **(I)** Bar graph displaying the proportions of cells with CloneIDs in each major cell population in the iE7.5^{Early2}-aE16.5 dataset, showing comparable proportions across cell types. **(J)** UpSet plot showing extensive clone sharing across germ layers (35%), indicating the presence of epiblast cells at the time of injection (early E7.5). The inset chord diagram illustrates the extent and direction of clonal sharing, with ribbon width proportional to the number of shared clones. **(K)** UMAP plot highlighting cells belonging to local or cross-region clones in the iE7.5^{Early2}-aE16.5 dataset. Notably, only ENS clones from germ-layer-pure clones showed dispersion across gut regions, replicating the findings from iE7.5-aE16.5 and iE7.5^{Early}-aE16.5 datasets presented in Figure 5B, E. (**L**) Heatmap showing the distribution of the 59 cross-region ENS clones in the iE7.5^{Early2}-aE16.5 dataset. (**M**) Heatmap showing the composition of ENS cell states/types in local and cross-region clones, revealing no apparent bias. **(N)** UMAP plot showing the differentiation states of ENS cells in clones with distinct regional dispersion patterns. (**O**) Violin plot showing comparable levels of *Pou3f3* expression in colonic ENS cells belonging to either local colonic clones or cross-region clones shared with ENS cells in the small intestine or stomach. Analyses in (**L-O**) were restricted to germ-layer pure clones. Panel (A) was created with Biorender.com.

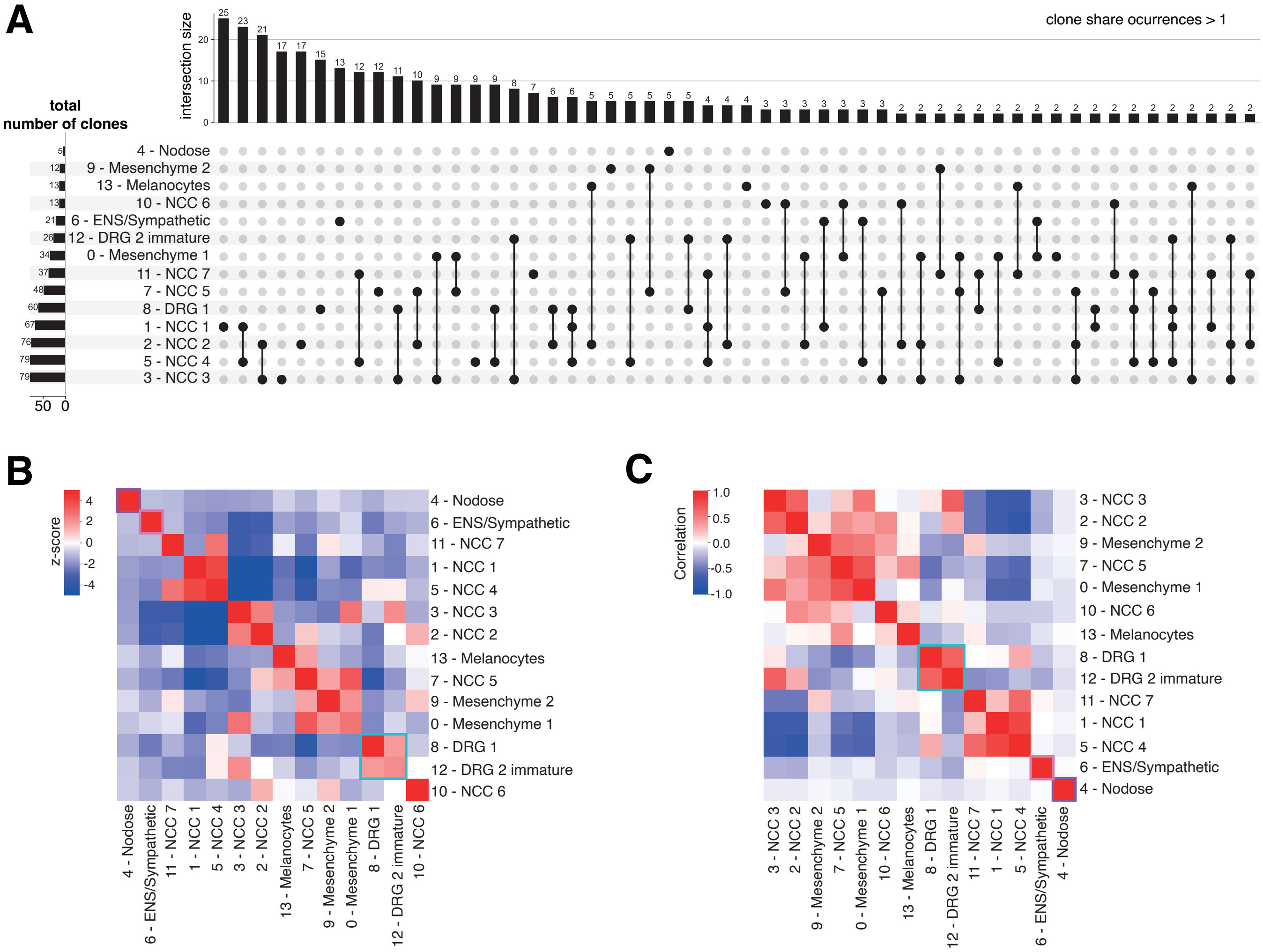

Fig. S10. Barcode sharing analysis of the E9.5-E10.5 neural crest/placode scRNA-seq dataset investigated in Figure 1. (**A**) UpSet plot showing number of clones shared within and between annotated cell populations in the E9.5-10.5 dataset (related to Figure 1). (**B**) Heatmap showing the clonal coupling z-scores, computed as deviations of observed clone sharing relative to randomized data for neural crest/placode cell populations in the E9.5-10.5 dataset. (**C**) Heatmap of correlation matrix of clonal coupling z-scores. Note that the nodose (dark purple), ENS/sympathetic (pink), and DRG clusters (turquise box) show low correlations with one another, suggesting that their primordia are segregated by E7.5.

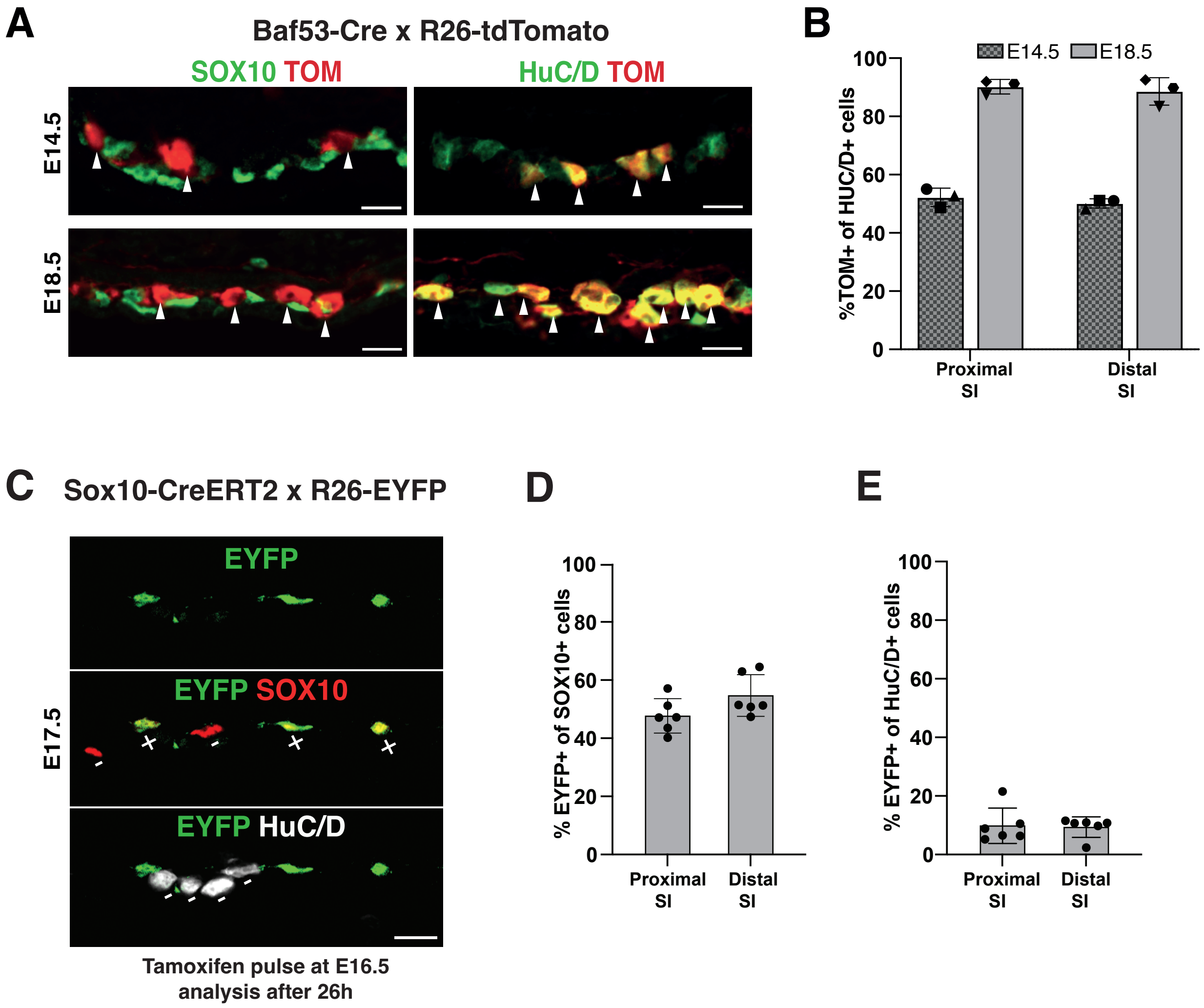

Fig. S11. Baf53b-Cre and Sox10-CreERT mice can be used to induce reporter expression in developing ENS neurons and progenitors/glia, respectively. (A) Representative confocal images of small intestine showing TOM expression in HuC/D+ neurons but not in SOX10+ glia/progenitors. TOM+ cells is indicated with white arrowheads. Scale bars: 20μm. **(B)** Bar graphs showing percentage of HuC/D+ cells expressing TOM as represented in (A). Bars represent mean ± SD. n=3 mice **(C)** Representative confocal images of small intestine showing that EYFP is predominantly induced in SOX10+ cells rather than HuC/D+ cells after 26 hours following tamoxifen administration in Sox10-CreERT2; R26-EYFP females at E16.5. (**D**) Bar graph showing percentage of SOX10 cells expressing EYFP as represented in (C). **(E)** Bar graph showing percentage of HuC/D cells expressing EYFP as represented in (C). Bars represent mean ± SD. n=6 mice. SI: small intestine.

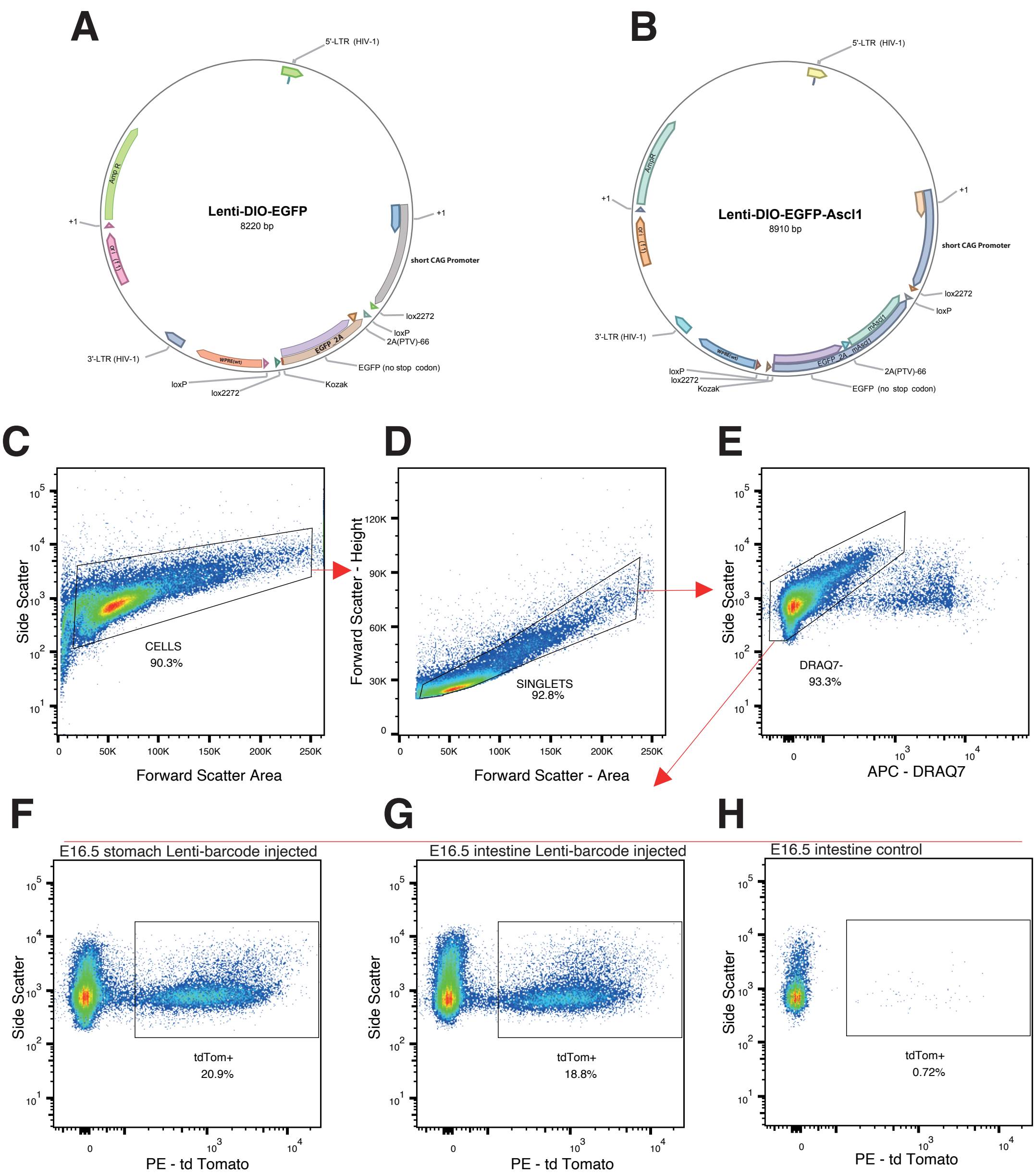

Fig. S12 Lentiviral vector design and flow cytometry gating strategy.
(A) Plasmid map of Lenti-DIO-EGFP, a third-generation HIV-1–based lentiviral vector driving Cre-dependent EGFP expression via a shortened CAG promoter. The bicistronic cassette contains EGFP, and a PTV-1 2A self-cleaving peptide, flanked by double-floxed inverted orientation (DIO) for Cre-dependent expression. Transgene stability is conferred by WPRE, with vector maintenance supported by an f1 ori, AmpR selection cassette, and polyadenylation signal within the backbone.**(B)** Plasmid map of LV-DIO-EGFP-Ascl1, which shares the same backbone as **(A)** but encodes a EGFP–P2A–mAscl1 cassette, enabling Cre-dependent co-expression of EGFP and the transcription factor mAscl1 as separate proteins via PTV-1 2A-mediated self-cleavage. **(C-H)** Representative Flow Cytometry Data plots showing gating strategy for sorting tdTomato+ cells from E16.5 mouse gut injected with LV-EF1A-H2B-tdTomato-30N. Forward vs Side Scatter plot gating to identify cells **(C)**, Side Scatter vs Trigger Pulse Width to exclude doublets and multiplets **(D)**. DRAQ7- gating to exclude unhealthy cells **(E)**. Red fluorescence (580/30) used to distinguish tdTomato+ cells from autofluorescent cells in stomach or intestine of an injected embryo **(F, G)**. Gating of a control intestine **(H)**.

Dataset legends:

Dataset S1: Table summarizing markers used to annotate scRNA-seq clusters with references.

Dataset S2: Table with summary statistics for the barcoded scRNA-sequencing datasets generated in this study (iE7.5-aE16.5, iE7.5^{Early}-aE16.5, iE7.5^{Early2}-aE16.5).

Dataset S3: Differentially Expressed Genes (DEGs) for clusters shown in Figure 1C. Clusters represents reprocessed neural crest/placode cells from E9.5 and E10.5 embryos injected at E7.5. For each gene, the following information is provided: cluster identity, gene name, log fold change (logfoldchanges), raw p-value (p_vals), and adjusted *p*-value based on Benjamini-Hochberg method (pvals_adj). Genes are ordered within each cluster by decreasing log fold change.

Dataset S4: Differentially Expressed Genes (DEGs) for clusters shown in Figure 2D. Clusters in the iE7.5-aE16.5 dataset. For each gene, the following information is provided: cluster identity, gene name, average log2 fold change (avg_log2FC), raw *p*-value (p_val), and adjusted *p*-value based on Bonferroni correction (p_val_adj). Additionally, difference in the percentage of expressing cells between the target cluster and all other cells (pct_diff = pct.1 − pct.2) is provided. Genes are ordered within each cluster by decreasing average log2 fold change.

Dataset S5: Differentially Expressed Genes (DEGs) for clusters shown in Figure 2E, F. Refined fibroblast and smooth muscle-like subclusters from the iE7.5-aE16.5 dataset. For each gene, the following information is provided: cluster identity, gene name, average log2 fold change (avg_log2FC), raw *p*-value (p_val), and adjusted *p*-value based on Bonferroni correction (p_val_adj). Additionally, difference in the percentage of expressing cells

between the target cluster and all other cells (pct_diff = pct.1 − pct.2) is provided. Genes are ordered within each cluster by decreasing average log2 fold change.

Dataset S6: Differentially Expressed Genes (DEGs) for clusters shown in Figure 3B. Major cell types after merging cluster belonging to same cell type in the iE7.5-aE16.5 dataset. For each gene, the following information is provided: cluster identity, gene name, average log2 fold change (avg_log2FC), raw *p*-value (p_val), and adjusted *p*-value based on Bonferroni correction (p_val_adj). Additionally, difference in the percentage of expressing cells between the target cluster and all other cells (pct_diff = pct.1 − pct.2) is provided. Genes are ordered within each cluster by decreasing average log2 fold change.

Dataset S7: Differentially Expressed Genes (DEGs) for clusters shown in Figure 4D. Refined clusters from the iE7.5^{Early}-aE16.5 dataset. For each gene, the following information is provided: cluster identity, gene name, average log2 fold change (avg_log2FC), raw *p*-value (p_val), and adjusted *p*-value based on Bonferroni correction (p_val_adj). Additionally, difference in the percentage of expressing cells between the target cluster and all other cells (pct_diff = pct.1 − pct.2) is provided. Genes are ordered within each cluster by decreasing average log2 fold change.

Dataset S8: Differentially Expressed Genes (DEGs) for clusters shown in SI Appendix, Fig. S9C. Refined clusters from the iE7.5^{Early2}-aE16.5 dataset. For each gene, the following information is provided: cluster identity, gene name, average log2 fold change (avg_log2FC), raw *p*-value (p_val), and adjusted *p*-value based on Bonferroni correction (p_val_adj). Additionally, difference in the percentage of expressing cells between the target cluster and all other cells (pct_diff = pct.1 − pct.2) is provided. Genes are ordered within each cluster by decreasing average log2 fold change.

SI References (for supplemental material and methods and Dataset S1):

1. C. Laranjeira *et al.*, Glial cells in the mouse enteric nervous system can undergo neurogenesis in response to injury. *J Clin Invest* **121**, 3412–3424 (2011).
2. S. de Haan *et al.*, Ectoderm barcoding reveals neural and cochlear compartmentalization. *Science* **388**, 60–68 (2025).
3. M. Ratz *et al.*, Clonal relations in the mouse brain revealed by single-cell and spatial transcriptomics. *Nat Neurosci* **25**, 285–294 (2022).
4. K. Mangold, J. He, S. Stokman, E. R. Andersson, Murine Neural Plate Targeting by In Utero Nano-Injection (NEPTUNE) at Embryonic Day 7.5. *J Vis Exp* 10.3791/63148 (2022).
5. K. Mangold *et al.*, Highly efficient manipulation of nervous system gene expression with NEPTUNE. *Cell Rep Methods* **1** (2021).
6. Y. Cao, X. Wang, G. Peng, SCSA: A Cell Type Annotation Tool for Single-Cell RNA-seq Data. *Front Genet* **11**, 490 (2020).
7. R. C. Bandler *et al.*, Single-cell delineation of lineage and genetic identity in the mouse brain. *Nature* **601**, 404–409 (2022).
8. D. E. Wagner *et al.*, Single-cell mapping of gene expression landscapes and lineage in the zebrafish embryo. *Science* **360**, 981–987 (2018).
9. A. Visel, C. Thaller, G. Eichele, GenePaint.org: an atlas of gene expression patterns in the mouse embryo. *Nucleic Acids Res* **32**, D552–556 (2004).
10. A. Chen *et al.*, Spatiotemporal transcriptomic atlas of mouse organogenesis using DNA nanoball-patterned arrays. *Cell* **185**, 1777–1792 e1721 (2022).
11. A. Beverdam, F. Meijlink, Expression patterns of group-I aristaless-related genes during craniofacial and limb development. *Mech Dev* **107**, 163–167 (2001).
12. M. E. Lucas, F. Muller, R. Rudiger, P. D. Henion, H. Rohrer, The bHLH transcription factor hand2 is essential for noradrenergic differentiation of sympathetic neurons. *Development* **133**, 4015–4024 (2006).
13. Q. Ma, Z. Chen, I. del Barco Barrantes, J. L. de la Pompa, D. J. Anderson, neurogenin1 is essential for the determination of neuronal precursors for proximal cranial sensory ganglia. *Neuron* **20**, 469–482 (1998).
14. J. F. Martin, E. N. Olson, Identification of a prx1 limb enhancer. *Genesis* **26**, 225–229 (2000).
15. A. Pattyn, X. Morin, H. Cremer, C. Goridis, J. F. Brunet, Expression and interactions of the two closely related homeobox genes Phox2a and Phox2b during neurogenesis. *Development* **124**, 4065–4075 (1997).
16. A. Pattyn, X. Morin, H. Cremer, C. Goridis, J. F. Brunet, The homeobox gene Phox2b is essential for the development of autonomic neural crest derivatives. *Nature* **399**, 366–370 (1999).
17. K. Radde-Gallwitz *et al.*, Expression of Islet1 marks the sensory and neuronal lineages in the mammalian inner ear. *J Comp Neurol* **477**, 412–421 (2004).

18. M. Schmidt *et al.*, The transcription factors AP-2beta and AP-2alpha are required for survival of sympathetic progenitors and differentiated sympathetic neurons. *Dev Biol* **355**, 89–100 (2011).
19. H. Yajima *et al.*, Six1 is a key regulator of the developmental and evolutionary architecture of sensory neurons in craniates. *BMC Biol* **12**, 40 (2014).
20. F. D'Autreaux, Y. Morikawa, P. Cserjesi, M. D. Gershon, Hand2 is necessary for terminal differentiation of enteric neurons from crest-derived precursors but not for their migration into the gut or for formation of glia. *Development* **134**, 2237–2249 (2007).
21. F. Guillemot *et al.*, Mammalian achaete-scute homolog 1 is required for the early development of olfactory and autonomic neurons. *Cell* **75**, 463–476 (1993).
22. Q. Ma, C. Fode, F. Guillemot, D. J. Anderson, Neurogenin1 and neurogenin2 control two distinct waves of neurogenesis in developing dorsal root ganglia. *Genes Dev* **13**, 1717–1728 (1999).
23. S. Nishikawa *et al.*, In utero manipulation of coat color formation by a monoclonal anti-c-kit antibody: two distinct waves of c-kit-dependency during melanocyte development. *EMBO J* **10**, 2111–2118 (1991).
24. K. Opdecamp *et al.*, Melanocyte development in vivo and in neural crest cell cultures: crucial dependence on the Mitf basic-helix-loop-helix-zipper transcription factor. *Development* **124**, 2377–2386 (1997).
25. K. P. Steel, D. R. Davidson, I. J. Jackson, TRP-2/DT, a new early melanoblast marker, shows that steel growth factor (c-kit ligand) is a survival factor. *Development* **115**, 1111–1119 (1992).
26. S. Vermeiren *et al.*, Prdm12 represses the expression of the visceral neuron determinants Phox2a/b in developing somatosensory ganglia. *iScience* **26**, 108364 (2023).
27. E. Blaugrund *et al.*, Distinct subpopulations of enteric neuronal progenitors defined by time of development, sympathoadrenal lineage markers and Mash-1-dependence. *Development* **122**, 309–320 (1996).
28. K. Morarach *et al.*, Diversification of molecularly defined myenteric neuron classes revealed by single-cell RNA sequencing. *Nat Neurosci* **24**, 34–46 (2021).
29. M. Aikawa *et al.*, Human smooth muscle myosin heavy chain isoforms as molecular markers for vascular development and atherosclerosis. *Circ Res* **73**, 1000–1012 (1993).
30. A. Armulik, G. Genove, C. Betsholtz, Pericytes: developmental, physiological, and pathological perspectives, problems, and promises. *Dev Cell* **21**, 193–215 (2011).
31. S. A. Baker *et al.*, Distribution and Ca(2+) signalling of fibroblast-like (PDGFR(+)) cells in the murine gastric fundus. *J Physiol* **591**, 6193–6208 (2013).

32. C. Bondjers *et al.*, Transcription profiling of platelet-derived growth factor-B-deficient mouse embryos identifies RGS5 as a novel marker for pericytes and vascular smooth muscle cells. *Am J Pathol* **162**, 721–729 (2003).
33. B. J. Bondow, M. L. Faber, K. J. Wojta, E. M. Walker, M. A. Battle, E-cadherin is required for intestinal morphogenesis in the mouse. *Dev Biol* **371**, 1–12 (2012).
34. M. B. Buechler *et al.*, Cross-tissue organization of the fibroblast lineage. *Nature* **593**, 575–579 (2021).
35. R. M. Farahani, M. Xaymardan, Platelet-Derived Growth Factor Receptor Alpha as a Marker of Mesenchymal Stem Cells in Development and Stem Cell Biology. *Stem Cells Int* **2015**, 362753 (2015).
36. P. J. Gomez-Pinilla *et al.*, Ano1 is a selective marker of interstitial cells of Cajal in the human and mouse gastrointestinal tract. *Am J Physiol Gastrointest Liver Physiol* **296**, G1370–1381 (2009).
37. S. E. Ha *et al.*, Transcriptome profiling of subepithelial PDGFRalpha cells in colonic mucosa reveals several cell-selective markers. *PLoS One* **17**, e0261743 (2022).
38. T. A. Heanue, V. Pachnis, Enteric nervous system development and Hirschsprung's disease: advances in genetic and stem cell studies. *Nat Rev Neurosci* **8**, 466–479 (2007).
39. R. Inoue, L. H. Kurahara, K. Hiraishi, TRP channels in cardiac and intestinal fibrosis. *Semin Cell Dev Biol* **94**, 40–49 (2019).
40. E. P. Kalabusheva *et al.*, A Kaleidoscope of Keratin Gene Expression and the Mosaic of Its Regulatory Mechanisms. *Int J Mol Sci* **24** (2023).
41. U. Lendahl, L. Muhl, C. Betsholtz, Identification, discrimination and heterogeneity of fibroblasts. *Nat Commun* **13**, 3409 (2022).
42. S. V. Litvinov, M. P. Velders, H. A. Bakker, G. J. Fleuren, S. O. Warnaar, Ep-CAM: a human epithelial antigen is a homophilic cell-cell adhesion molecule. *J Cell Biol* **125**, 437–446 (1994).
43. H. Moon, J. Zhu, L. R. Donahue, E. Choi, A. C. White, Krt5(+)/Krt15(+) foregut basal progenitors give rise to cyclooxygenase-2-dependent tumours in response to gastric acid stress. *Nat Commun* **10**, 2225 (2019).
44. P. J. Newman, The biology of PECAM-1. *J Clin Invest* **100**, S25–29 (1997).
45. Y. Rinkevich *et al.*, Identification and prospective isolation of a mesothelial precursor lineage giving rise to smooth muscle cells and fibroblasts for mammalian internal organs, and their vasculature. *Nat Cell Biol* **14**, 1251–1260 (2012).
46. S. Schneider *et al.*, Single Nucleus Sequencing of Human Colon Myenteric Plexus-Associated Visceral Smooth Muscle Cells, Platelet Derived Growth Factor Receptor Alpha Cells, and Interstitial Cells of Cajal. *Gastro Hep Adv* **2**, 380–394 (2023).
47. M. Shoshkes-Carmel *et al.*, Subepithelial telocytes are an important source of Wnts that supports intestinal crypts. *Nature* **557**, 242–246 (2018).
48. T. Tabib *et al.*, Myofibroblast transcriptome indicates SFRP2(hi) fibroblast progenitors in systemic sclerosis skin. *Nat Commun* **12**, 4384 (2021).

49. M. Torres, E. Gomez-Pardo, P. Gruss, Pax2 contributes to inner ear patterning and optic nerve trajectory. *Development* **122**, 3381–3391 (1996).
50. I. S. Trowbridge, M. L. Thomas, CD45: an emerging role as a protein tyrosine phosphatase required for lymphocyte activation and development. *Annu Rev Immunol* **12**, 85–116 (1994).
51. S. E. Viitala E.W, Miihkinen M, Chalkidi N, Stavropoulou A, Lemmetyinen TT, Päivinen P, Kaptrio T, Hagström J, Haglund C, Mäkelä TP, Aittokallio T, Katajisto P, Koliaraki V, Ollila S. (2025) High-resolution integrative analysis allows characterization and spatial annotation of normal and cancer-associated colon fibroblasts. (bioRxiv).
52. S. M. Ward, A. J. Burns, S. Torihashi, S. C. Harney, K. M. Sanders, Impaired development of interstitial cells and intestinal electrical rhythmicity in steel mutants. *Am J Physiol* **269**, C1577–1585 (1995).
53. Q. Xu *et al.*, SMOC2 (high) myofibroblastic cancer-associated fibroblast drives primary cilia-associated tumor microenvironment remodeling and poor prognosis in gastric cancer. *Chin J Cancer Res* **37**, 603–623 (2025).
54. L. Yu, C. Qiu, R. Chen, A narrative review of research advances in the study of molecular markers of airway smooth muscle cells. *Ann Transl Med* **10**, 375 (2022).
55. K. Chang, I. Pastan, Molecular cloning of mesothelin, a differentiation antigen present on mesothelium, mesotheliomas, and ovarian cancers. *Proc Natl Acad Sci U S A* **93**, 136–140 (1996).
56. M. Kanamori-Katayama *et al.*, LRRN4 and UPK3B are markers of primary mesothelial cells. *PLoS One* **6**, e25391 (2011).
57. A. W. Langerak *et al.*, Expression of the Wilms' tumor gene WT1 in human malignant mesothelioma cell lines and relationship to platelet-derived growth factor A and insulin-like growth factor 2 expression. *Genes Chromosomes Cancer* **12**, 87–96 (1995).
58. N. Nagy, A. M. Goldstein, Enteric nervous system development: A crest cell's journey from neural tube to colon. *Semin Cell Dev Biol* **66**, 94–106 (2017).
59. M. Barrenschee *et al.*, SNAP-25 is abundantly expressed in enteric neuronal networks and upregulated by the neurotrophic factor GDNF. *Histochem Cell Biol* **143**, 611–623 (2015).
60. T. Uesaka, M. Nagashimada, H. Enomoto, Neuronal Differentiation in Schwann Cell Lineage Underlies Postnatal Neurogenesis in the Enteric Nervous System. *J Neurosci* **35**, 9879–9888 (2015).
61. P. B. Wright *et al.*, The mannose receptor (CD206) identifies a population of colonic macrophages in health and inflammatory bowel disease. *Sci Rep* **11**, 19616 (2021).
62. S. De Schepper *et al.*, Self-Maintaining Gut Macrophages Are Essential for Intestinal Homeostasis. *Cell* **175**, 400–415 e413 (2018).
63. M. M. Davis, Y. H. Chien, N. R. Gascoigne, S. M. Hedrick, A murine T cell receptor gene complex: isolation, structure and rearrangement. *Immunol Rev* **81**, 235–258 (1984).
